# Recurrent moderate hypoglycemia accelerates the progression of Alzheimer’s disease through impairment of the TRPC6/GLUT3 pathway

**DOI:** 10.1172/jci.insight.154595

**Published:** 2022-03-08

**Authors:** Chengkang He, Qiang Li, Yuanting Cui, Peng Gao, Wentao Shu, Qing Zhou, Lijuan Wang, Li Li, Zongshi Lu, Yu Zhao, Huan Ma, Xiaowei Chen, Hongbo Jia, Hongting Zheng, Gangyi Yang, Daoyan Liu, Martin Tepel, Zhiming Zhu

**Affiliations:** 1 Department of Hypertension and Endocrinology, Center for Hypertension and Metabolic Diseases, Daping Hospital, Army Medical University, Chongqing Institute of Hypertension, Chongqing Institute for Brain and Intelligence, Chongqing, China.; 2 Brain Research Center, Army Medical University, Chongqing Institute for Brain and Intelligence, Chongqing, China.; 3 Suzhou Institute of Biomedical Engineering and Technology, Chinese Academy of Sciences, Suzhou, China.; 4 Department of Endocrinology, Translational Research Key Laboratory for Diabetes, Xinqiao Hospital, Army Medical University, Chongqing, China.; 5 Endocrine Department, Second Affiliated Hospital of Chongqing Medical University, Chongqing, China.; 6 Odense University Hospital, Department of Nephrology, University of Southern Denmark, Institute for Molecular Medicine, Cardiovascular and Renal Research, Institute of Clinical Research, Odense, Denmark.

**Keywords:** Endocrinology, Alzheimer disease, Diabetes

## Abstract

Currently, the most effective strategy for dealing with Alzheimer’s disease (AD) is delaying the onset of dementia. Severe hypoglycemia is strongly associated with dementia; however, the effects of recurrent moderate hypoglycemia (RH) on the progression of cognitive deficits in patients with diabetes with genetic susceptibility to AD remain unclear. Here, we report that insulin-controlled hyperglycemia slightly aggravated AD-type pathologies and cognitive impairment; however, RH significantly increased neuronal hyperactivity and accelerated the progression of cognitive deficits in streptozotocin-induced (STZ-induced) diabetic APP/PS1 mice. Glucose transporter 3–mediated (GLUT3-mediated) neuronal glucose uptake was not significantly altered under hyperglycemia but was markedly reduced by RH, which induced excessive mitochondrial fission in the hippocampus. Overexpression of GLUT3, specifically in the dentate gyrus (DG) area of the hippocampus, enhanced mitochondrial function and improved cognitive deficits. Activation of the transient receptor potential channel 6 (TRPC6) increased GLUT3-mediated glucose uptake in the brain and alleviated RH-induced cognitive deficits, and inactivation of the Ca^2+^/AMPK pathway was responsible for TRPC6-induced GLUT3 inhibition. Taken together, RH impairs brain GLUT3-mediated glucose uptake and further provokes neuronal mitochondrial dysfunction by inhibiting TRPC6 expression, which then accelerates progression of cognitive deficits in diabetic APP/PS1 mice. Avoiding RH is essential for glycemic control in patients with diabetes, and TRPC6/GLUT3 represents potent targets for delaying the onset of dementia in patients with diabetes.

## Introduction

Alzheimer’s disease (AD) is the most common form of dementia and is characterized by progressive deficits in memory and cognitive function. Clinically, AD may be early-onset familial AD or late-onset sporadic AD, with the latter closely associated with genetic and metabolic risk factors, such as diabetes, hypertension, obesity, smoking, and depression, that significantly exacerbate AD-type pathologies and promote disease progression (1–3). Efficient therapies remain elusive despite intensive research on treatments for AD over the past few decades. Given that the symptoms and pathology of late-onset sporadic AD develop over many years or even decades, control of risk factors is essential to delay or prevent the progression of severe cognitive deficits.

Nevertheless, determinants of accelerated cognitive decline in patients with diabetes with genetic susceptibility to AD are less clear because glucose dyshomeostasis is complex and can range from chronic hyperglycemia to treatment-induced recurrent hypoglycemia or be combined with hypertension and cardiovascular complications. Hypoglycemic episodes due to tight glycemic control using insulin or other hypoglycemic agents occur in approximately 25%–30% of patients with diabetes (4, 5). Previous studies have demonstrated that even 1 episode can induce hypoglycemia-associated autonomic failure and subsequent recurrent hypoglycemia (6, 7). Moderate hypoglycemia (blood glucose falls to 3.3–2.2 mM) is far more common than severe hypoglycemia (5, 8), and patients with diabetes with a history of severe hypoglycemia are at higher risk of cognitive impairment and AD (9, 10). However, whether recurrent moderate hypoglycemia (RH) is related to AD progression remains unknown because RH is usually self-remitted yet not easily discernible. Interestingly, while RH has been reported to exert protective effects on cognition in normal rats and in animals with a history of severe hypoglycemia (11, 12), contradictory effects were seen in diabetic mice (13), implying heterogeneity in the effects of RH on cognition and reiterating the need to delineate the underlying mechanism(s) to delay onset of dementia in patients with diabetes susceptible to AD.

Aerobic oxidation of glucose in the mitochondria is the predominant source of brain ATP. While translocation of glucose across the blood-brain barrier (BBB) and astrocyte plasma membrane is mainly mediated by glucose transporter 1 (GLUT1) (14, 15), glucose uptake into neurons is almost exclusively mediated by GLUT3 (16–18), and the expression of other glucose transporters is much lower than that of either (19). Importantly, glucose transporter expression significantly changes in response to energy demands or under pathophysiological conditions; indeed, brain GLUT1 and GLUT3 protein levels are considerably lower in patients with AD and animal models of AD (20). In STZ-induced diabetic rats, GLUT3 protein expression was specifically upregulated in the hippocampus, whereas GLUT1 protein abundance was similar to that of nondiabetic littermates (21, 22). High-fat diet feeding of mice downregulates GLUT1 expression in BBB (23). As GLUT3-mediated glucose transport into neurons is metabolized in mitochondria to produce ATP, and mitochondrial dysfunction is causally associated with dementia (2, 24, 25), the question is whether RH can affect the expression and function of brain GLUTs and further impair brain mitochondrial function.

The transient receptor potential channel 6 (TRPC6) is a Ca^2+^-permeable nonselective cation channel whose expression is sensitive to glucose fluctuation (26). Animal experiments and clinical studies showed that neuronal TRPC6 dysfunction is closely associated with the pathogenesis of AD (27, 28). We have recently shown that a RH-induced reduction in TRPC6 expression led to cognitive impairment under diabetic conditions by impairing hippocampal mitochondrial function (26). However, it is unknown whether RH similarly promotes the development of cognitive deficits in diabetic APP/PS1 (APP/PS1-DM) mice. As the expression of GLUTs is markedly regulated by free cytosolic Ca^2+^ and AMPK (29–31), we hypothesized that TRPC6 dysfunction could affect GLUT-mediated glucose uptake. Therefore, this study examined if and how RH participates in the progression of AD-type pathology and cognitive deficits in APP/PS1-DM mice.

## Results

### RH increases neuronal hyperactivity in APP/PS1-DM mice.

Parameters, such as body weight, blood pressure, random blood glucose (RBG), glucose tolerance, and HbA1c were not significantly different between the APP/PS1-DM and APP/PS1-DM-RH mice (Supplemental Figure 1, A–E; supplemental material available online with this article; https://doi.org/10.1172/jci.insight.154595DS1). STZ-induced diabetes significantly reduced body weight and glucose tolerance and increased RBG and HbA1c levels (Supplemental Figure 1, A, C, and E), but did not significantly alter blood pressure when compared with APP/PS1 mice (Supplemental Figure 1B). There were no significant differences between WT and APP/PS1 groups (Supplemental Figure 1, A–E). During insulin-induced hypoglycemic episodes, blood glucose in the APP/PS1-DM-RH group ranged between 2.2–3.9 mM, which was sustained for about 120 minutes (Supplemental Figure 1F).

**Figure 1:**
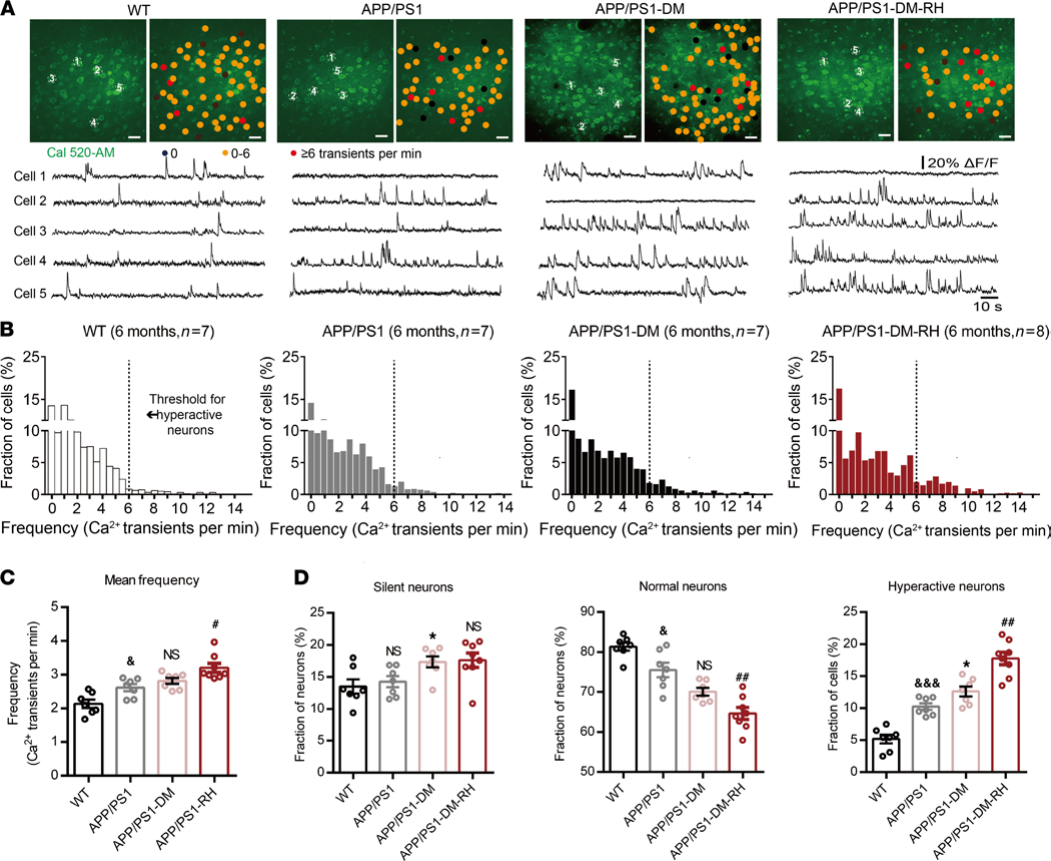
Neuronal hyperactivity in APP/PS1-DM mice was significantly increased by RH. (**A**) Top, representative in vivo 2-photon Ca^2+^ images of Cal 520 loading (green) layer 2/3 neurons in the prefrontal cortex from a 6-month-old WT, APP/PS1, STZ-induced APP/PS1-DM, and STZ-induced APP/PS1-DM-RH mice. Neurons were color-coded according to their mean activity. Black, silent neurons (0 transients/min); orange, normal neurons; red, hyperactive neurons (≥6 transients/min). Bottom, spontaneous Ca^2+^ transients of soma indicated in the top panel. (**B**) Frequency distributions of recorded neurons from WT (*n* = 417 neurons in 7 mice), APP/PS1 (*n* = 376 neurons in 7 mice), APP/PS1-DM (*n* = 411 neurons in 7 mice), and APP/PS1-DM-RH (*n* = 446 neurons in 8 mice) mice. The dashed line serves as the threshold for hyperactive neurons. (**C**) Mean neuronal frequencies for WT (2.136 ± 0.1226 transients/min), APP/PS1 (2.571 ± 0.1177 transients/min), APP/PS1-DM (2.814 ± 0.08978 transients/min), and APP/PS1-DM-RH (3.199 ± 0.1310 transients/min). (**D**) Fraction of silent, normal, and hyperactive neurons from indicated groups. The data are expressed as the mean ± SEM. The differences between groups were assessed by 1-way ANOVA followed by Dunnett’s multiple comparisons test. *P < 0.05, APP/PS1-DM versus APP/PS1; ^&^*P* < 0.05 and ^&&&^*P* < 0.001, WT versus APP/PS1*;* ^#^*P* < 0.05 and ^##^*P* < 0.01, APP/PS1-DM versus APP/PS1-DM-RH; APP/PS1 versus APP/PS1-DM; NS, no significant difference. Scale bars, 200 µm.

Spontaneous ongoing neuronal activity is known to be a significant determinant of brain information processing (32, 33), and disturbances in neuronal activity are one of the main functional defects in animal models of AD (34). Here, we employed in vivo 2-photon Ca^2+^ imaging of layer 2/3 neurons in the prefrontal cortex (PFC) to reveal the effects of RH on neuronal activity and show increased frequency of Ca^2+^ transients and a greater fraction of hyperactive neurons in 6-month-old APP/PS1 mice compared with WT mice, indicating neuronal hyperactivity without changes in the fraction of silent neurons (Figure 1, A–D). In APP/PS1-DM mice, the fraction of both silent and hyperactive neurons was higher compared with APP/PS1 mice (Figure 1D). Next, we assessed spontaneous ongoing neuronal activity in APP/PS1-DM-RH mice and found that RH significantly increased the frequency of Ca^2+^ transients (APP/PS1-DM versus APP/PS1-DM-RH, 2.80 ± 0.09 versus 3.20 ± 0.13 transients/min, respectively) and the fraction of hyperactive neurons (12.60 ± 0.77 (%) versus 17.76 ± 0.97 (%), respectively; Figure 1, C and D). However, there was no significant difference in the fraction of silent neurons (Figure 1D). Taken together, these results reveal that RH induces neuronal hyperactivity in APP/PS1-DM mice.

### RH accelerates progression of AD-type pathologies and cognitive deficits.

**Figure 2:**
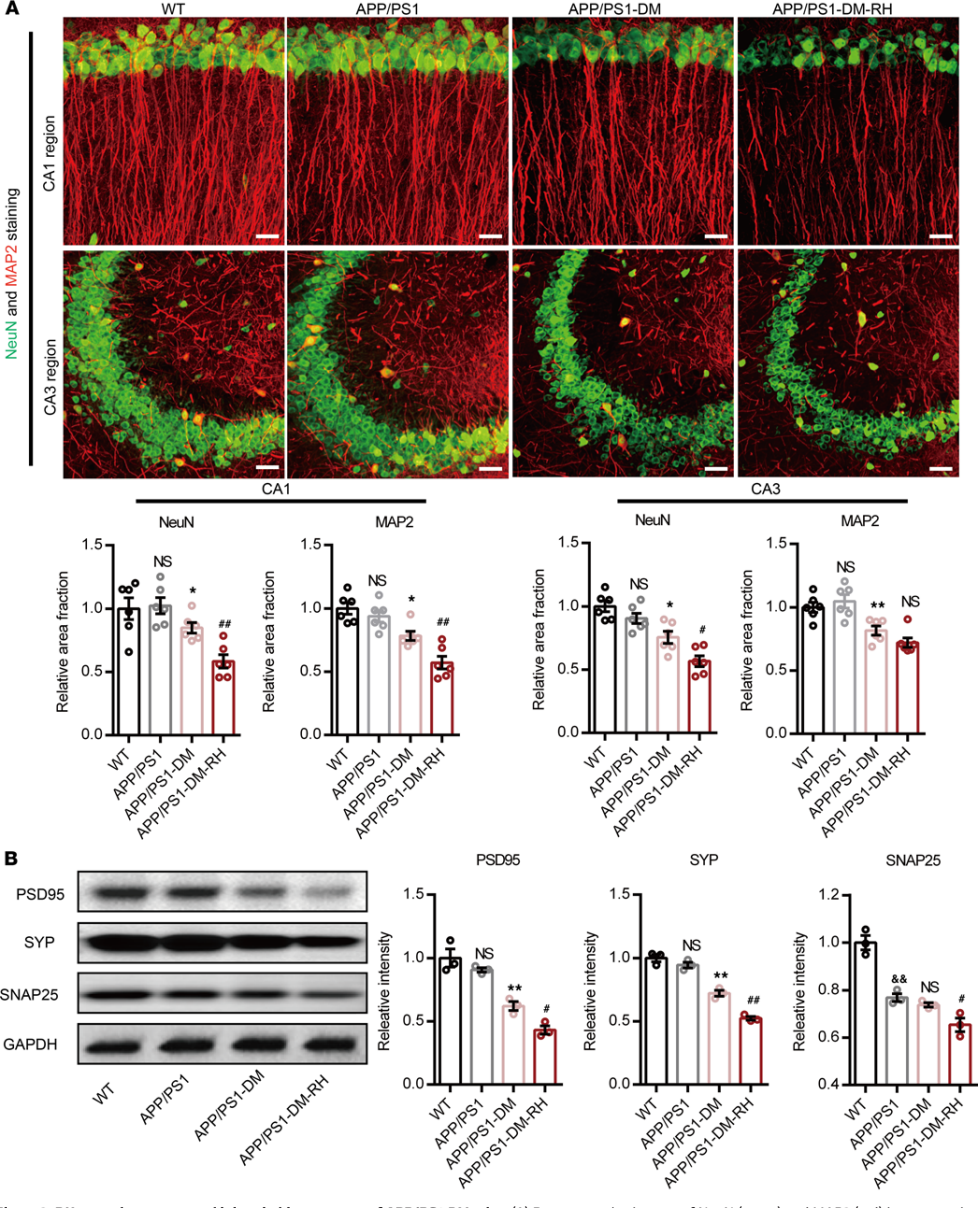
RH exacerbates neuronal injury in hippocampus of APP/PS1-DM mice. (A) Representative images of NeuN (green) and MAP2 (red) immunostaining in CA1 (top) and CA3 (bottom) region of hippocampus in WT, APP/PS1, APP/PS1-DM, and APP/PS1-DM-RH mice. Scale bar: 100 μm. Quantitative results are shown in the bottom of images (*n* = 4 mice for each group). (**B**) Western blot and quantitation for synapse-associated proteins including PSD95, SYP, and SNAP25 in hippocampal homogenates (*n* = 3 mice for each group). The data are expressed as the mean ± SEM. Statistical significance was assessed using unpaired Student’s *t* test. *P < 0.05 and **P < 0.01, APP/PS1-DM versus APP/PS1; ^#^*P* < 0.05 and ^##^*P* < 0.01, APP/PS1-DM versus APP/PS1-DM-RH; ^&&^*P* < 0.01, WT versus APP/PS1; WT versus APP/PS1; NS, no significant difference.

To investigate the direct effect of RH on neuronal morphology, we detected the expression level of neuronal and synaptic markers in the hippocampus. Compared with WT mice, 6-month-old APP/PS1 mice displayed only mild synaptic loss, as evidenced by negligible differences in the positive area fraction of NeuN and MAP2 staining (Figure 2A), or an abundance of PSD95 and synaptophysin (SYP) proteins, despite a reduction in synaptosomal-associated protein 25 (SNAP25) expression (Figure 2B). However, hippocampal neuronal and synaptic loss in STZ-induced APP/PS1-DM mice was prominent (Figure 2, A and B). Importantly, the positive area fraction of NeuN and MAP2, along with expression of PSD95, SYP, and SNAP25 proteins, were significantly lower in APP/PS1-DM-RH mice than in APP/PS1-DM mice (Figure 2, A and B), indicating that RH significantly aggravates neuronal and dendritic loss.

Next, we evaluated the effects of RH on brain Aβ protein deposition and neuroinflammation. Compared with WT mice, 6-month-old APP/PS1 mice displayed obvious Aβ protein deposition and neuroinflammation in the hippocampus, which was significantly exacerbated by RH in STZ-induced APP/PS1-DM mice (Supplemental Figure 2, A–D). Specifically, the total number of amyloid plaques, detected by 6E10 staining, were significantly increased by RH (Supplemental Figure 2A). ELISA results also indicated a significant increase in the level of Aβ40 and Aβ42 from APP/PS1-DM-RH mice (Supplemental Figure 2C). Astrocytosis and microgliosis were dramatically enhanced by RH (Supplemental Figure 2B), as were levels of proinflammatory cytokines, including TNF-α, IFN-γ, IL-1β, and IL-6, in hippocampal homogenates (Supplemental Figure 2D). These results demonstrate that RH markedly accelerates the progression of AD-type pathologies.

**Figure 3:**
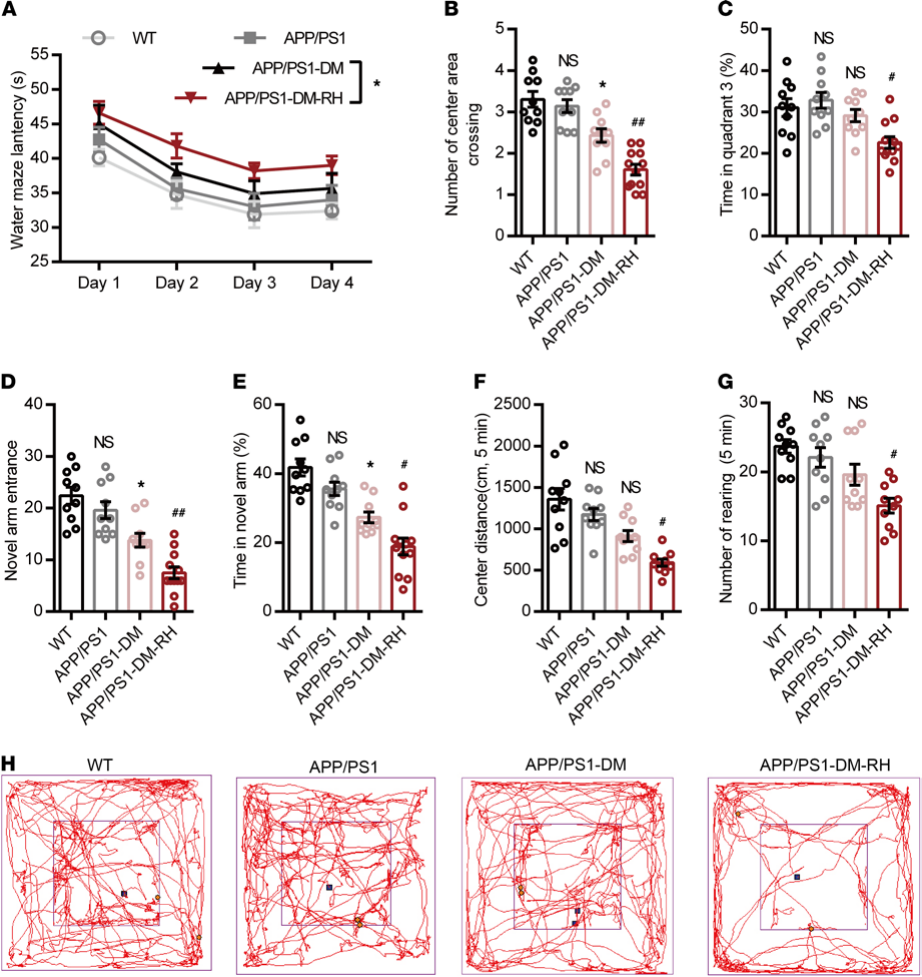
RH aggravates the impairment of behavioral performance in APP/PS1-DM mice. (**A**–**C**) Morris water maze test. (**A**) Escape latency during platform trials, (**B**) number of center area (where the hidden platform had previously been located) crossings, and (**C**) time spent in quadrant 3 (Q3) of water maze in probe test. (**D**) Novel arm entrance and (**E**) time spent in the Novel arm in Y-maze test. (**F**) Distance traveled in center region, (**G**) number of rearing, and (**H**) representative tracing graphs in the open-field test. *n* = 10–12 mice for each group. The data are expressed as the mean ± SEM. Statistical significance was assessed using 1-way ANOVA in **B**–**G** and 2-way ANOVA in A followed by Dunnett’s multiple-comparison test. *P < 0.05, APP/PS1-DM versus APP/PS1; ^#^*P* < 0.05 and ^##^*P* < 0.01, APP/PS1-DM versus APP/PS1-DM-RH; WT versus APP/PS1; NS, no significant difference.

Given these negative effects on neuronal activity and AD-type pathologies, we further investigated the cognitive sequelae due to RH in STZ-induced APP/PS1-DM mice using behavioral tests. WT and 6-month-old APP/PS1 mice performed equally well, while STZ-induced diabetes significantly impaired the performance of APP/PS1 mice (Figure 3, A–H). Importantly, the cognitive deficits in STZ-induced APP/PS1-DM mice were further aggravated by RH. In the Morris water maze test, APP/PS1-DM-RH mice required a longer latency escape (Figure 3A). The number of center area crossings (Figure 3B) and time spent in quadrant 3 (Figure 3C) by APP/PS1-DM-RH mice were significantly lower than that of APP/PS1-DM mice. In the Y-maze and open-field tests, APP/PS1-DM-RH mice performed worse than the APP/PS1-DM mice, as reflected by fewer entries, less time spent in the novel arm (Figure 3, D and E), shorter distance traveled in the center region, and reduced rearing (Figure 3, F–H). Additionally, we assessed the effects of RH on cognitive function in nondiabetic APP/PS1 mice and the results showed that RH treatment significantly impaired the performances of mice in behavioral tests (Supplemental Figure 3, A–G). These results indicate that RH significantly promotes the progression of cognitive deficits and accelerates the appearance of the dementia phenotype under diabetic condition.

### RH exacerbates brain mitochondrial dysfunction and energy stress.

**Figure 4:**
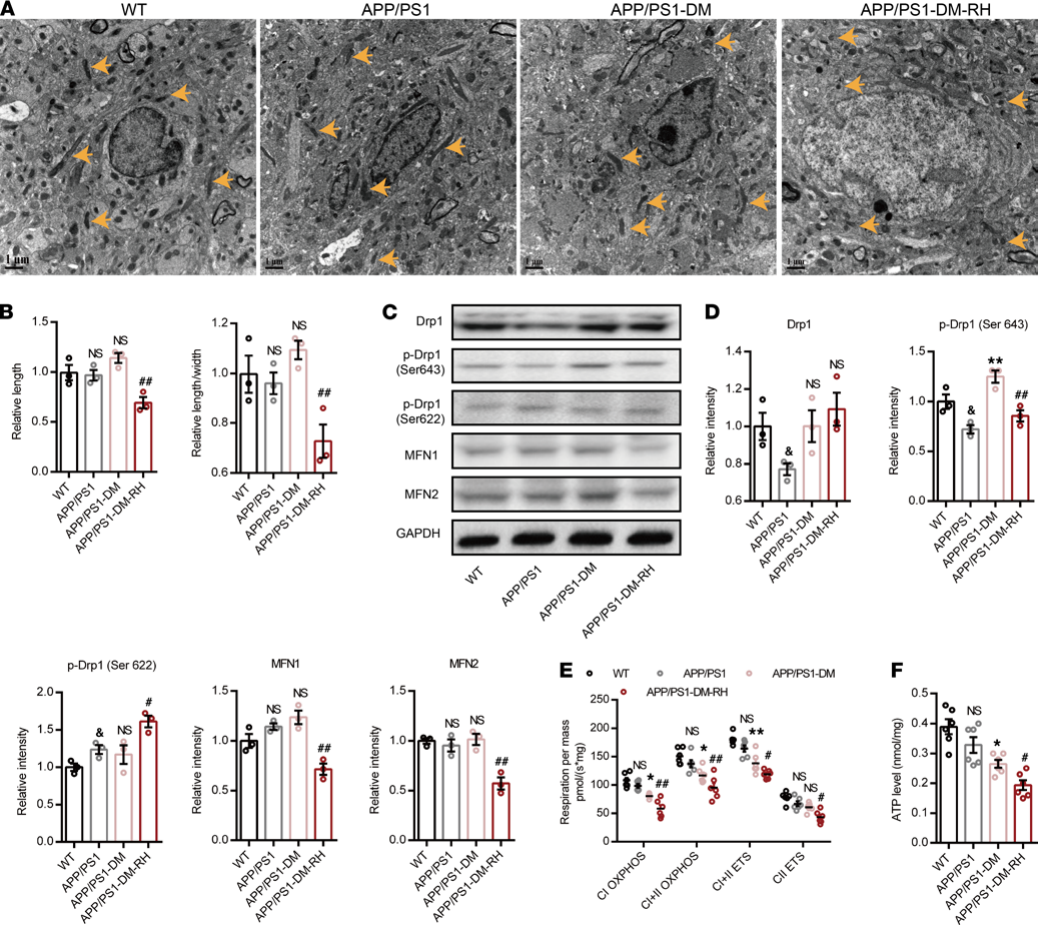
RH exacerbates brain mitochondrial dysfunction and energy stress in STZ-induced APP/PS1-DM mice. (**A**) TEM images showing the mitochondrial morphology (indicated by yellow arrows) in CA1 region of hippocampus. (**B**) Quantitative data of mitochondrial length and ratio of length to width (*n* = 200–300 mitochondria, 3 mice for each group). (**C) **Western blot images and (**D**) quantitation for Drp1, MFN1, MFN2, p-Drp1 (Ser 643), and p-Drp1 (Ser 622) in hippocampal homogenates (*n* = 3 mice for each group). (**E**) High-resolution respirometry measured the oxygen consumption capacity of hippocampal mitochondria (*n* = 6 mice for each group). CI OXPHOS, complex I oxidative phosphorylation capacity; CI+CII OXPHOS, complex I plus II oxidative phosphorylation capacity; CII ETS, complex II electron transfer system capacity; CI+CII ETS, complex I plus II electron transfer system capacity. (**F**) ATP content of hippocampus (*n* = 6 mice for each group). The data are expressed as the mean ± SEM. Statistical significance was assessed using unpaired Student’s *t* test in D, 1-way ANOVA in **B** and **F**, and 2-way ANOVA in **E** followed by Dunnett’s multiple-comparison test. ^&^*P* < 0.05, WT versus APP/PS1; *P < 0.05 and **P < 0.01, APP/PS1-DM versus APP/PS1; ^#^*P* < 0.05 and ^##^*P* < 0.01, APP/PS1-DM-RH versus APP/PS1-DM. NS, no significant difference. Scale bars: 1 µm.

As recurrent glucose deficiency may impair mitochondrial function and ultimately induce energy stress, we used transmission electron microscopy (TEM) and O2K to observe the morphology and function of hippocampal mitochondria and demonstrate that mitochondrial morphology and function, along with ATP content, were similar in 6-month-old APP/PS1 mice and WT mice (Figure 4, A–F). STZ-induced diabetes only slightly impaired mitochondrial morphology, but it increased expression of phosphorylated-dynamin-related protein 1 (p-Drp1) (Ser643) and markedly impaired both mitochondrial function and ATP content (Figure 4, A–F). Importantly, RH led to obvious fragmentation in the hippocampus, as reflected by the reduction in mitochondrial length (major axes) and the ratio of length to width (Figure 4, A and B). Consistently, the expression of p-Drp1 (Ser 643), MFN1, and MFN2 were significantly reduced while the expression of p-Drp1 (Ser 622) was increased in APP/PS1-DM-RH mice compared with APP/PS1-DM mice, suggesting excessive mitochondrial fission (Figure 4, C and D). Correspondingly, RH significantly exacerbated the reduction in mitochondrial oxidative phosphorylation and ATP content (Figure 4, E and F). Thus, these results demonstrate that RH exacerbates brain mitochondrial dysfunction and energy stress in STZ-induced APP/PS1-DM mice.

### RH reduces GLUT3-mediated glucose uptake in neurons.

**Figure 5:**
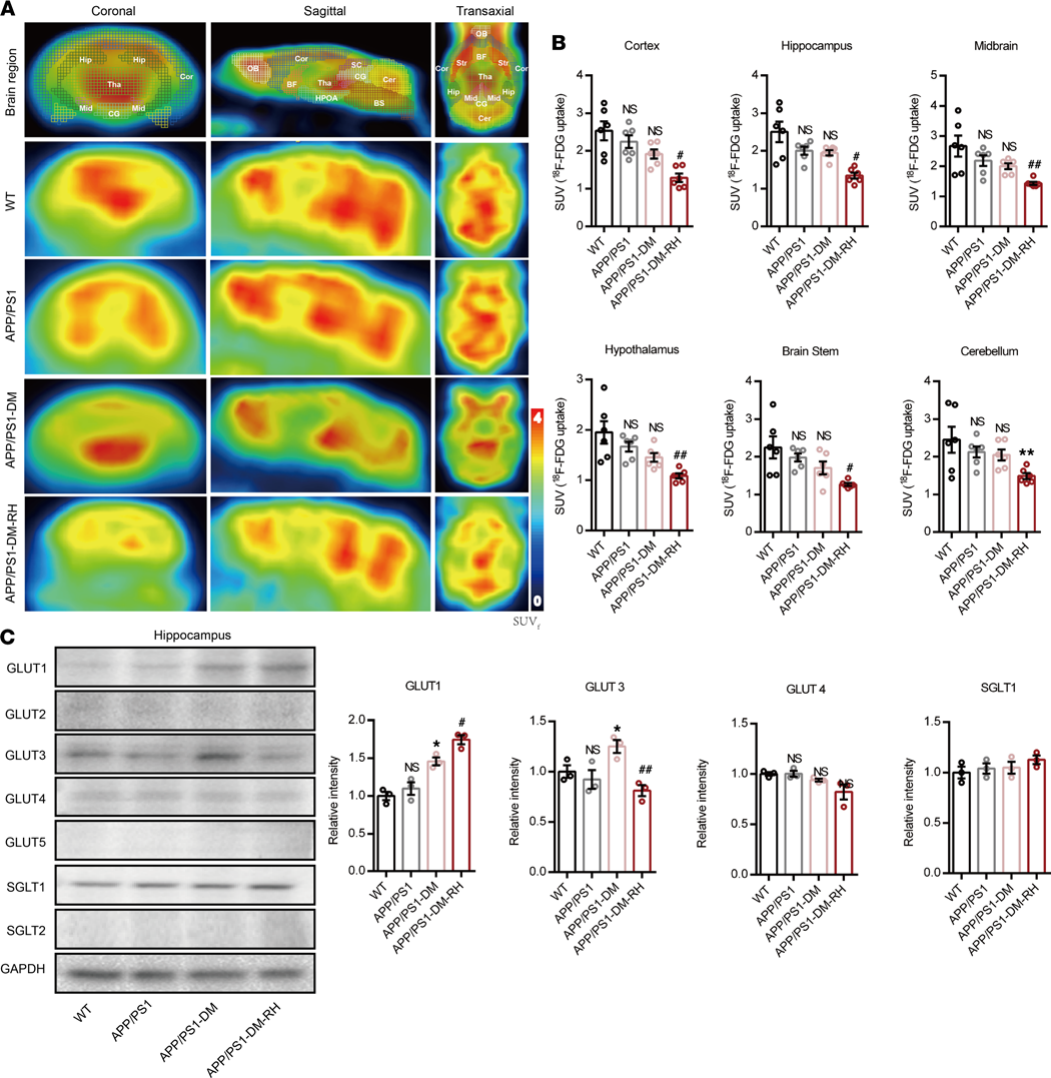
RH reduced brain GLUT3-mediated glucose uptake in STZ-induced APP/PS1-DM mice. (**A**) Representative PET/CT images showing in vivo brain ^18^F-FDG uptake (coronal, sagittal, and transaxial sections, *n* = 6 mice for each group). Cor, Cortex; Hip, hippocampus; Mid, midbrain; HPOA, hypothalamus; BS, brain stem; Cer, cerebellum; OB, Olfactory Bulb; BF, basal forebrain; Tha, thalamus; SC, superior colliculi; CG, central gray; Str, striatum. (**B**) Quantification for SUV of ^18^F-FDG in brain region (*n* = 6 for mice for each group). (**C**) Western blot and quantitation for GLUT1, GLUT2, GLUT3, GLUT4, GLUT5, SGLT1, and SGLT2 in hippocampal homogenates (*n* = 3 mice for each group). The data are expressed as the mean ± SEM. Statistical significance was assessed using unpaired Student’s *t* test. *P < 0.05 and **P < 0.01, APP/PS1-DM versus APP/PS1; ^#^*P* < 0.05 and ^##^*P* < 0.01, APP/PS1-DM-RH versus APP/PS1-DM; WT versus APP/PS1; NS, no significant difference.

Next, we investigated the mechanisms underlying such accelerated progression of cognitive deficits in APP/PS1-DM-RH mice. As transporter-mediated brain glucose uptake is the major source of energy for normal neuronal function, we assessed the effect of RH on brain glucose uptake by measuring the standard uptake value (SUV) of ^18^F-fluorodeoxyglucose (^18^F-FDG) in mice using micro-PET/CT. Interestingly, STZ-induced APP/PS1-DM mice showed nearly preserved SUV for ^18^F-FDG as 6-month-old APP/PS1 mice (Figure 5, A and B, and Supplemental Figure 4, A–H). However, the SUV was significantly lower after RH in the entire brain of STZ-induced APP/PS1-DM mice (Figure 5, A and B, and Supplemental Figure 4, A–H) and of nondiabetic APP/PS1 mice (Supplemental Figure 5, B–O), suggesting that RH impairs brain glucose uptake.

We further sought to identify the glucose transporter that contributes to this dysfunction and show that hippocampal expression of GLUT1 and GLUT3 was not changed significantly in APP/PS1 mice compared with WT mice; however, it was dramatically increased in APP/PS1-DM mice. Interestingly, the expression of GLUT1 was further increased while that of GLUT3 was significantly decreased in the APP/PS1-DM-RH group (Figure 5C). The expression of GLUT3 was also reduced in APP/PS1-RH mice compared with APP/PS1 mice (Supplemental Figure 5A). Immunofluorescent staining showed that the GLUT3 expression in the hippocampal CA1, CA3, and DG regions were significantly increased in APP/PS1-DM mice, while reduced by RH (Supplemental Figure 6, A–C). Expression of other glucose transporters such as sodium-glucose cotransporter 1 (SGLT1), SGLT2, GLUT2, GLUT4, and GLUT5 in the hippocampus either remained unchanged or were not detected after RH (Figure 5C), indicating that a RH-induced reduction in GLUT3 expression contributes to the impairment of neuronal glucose uptake.

### GLUT3 overexpression improves RH-induced mitochondrial dysfunction and cognitive deficits.

To uncover whether hippocampal mitochondrial dysfunction and cognitive deficits caused by RH causally result from the reduction of GLUT3-mediated glucose uptake, we first overexpressed GLUT3 in PC12 cells (Supplemental Figure 7, A and B) using lentiviral vector and found that it led to significantly increased mitochondrial function and ATP content (Supplemental Figure 7, C and D).

**Figure 6:**
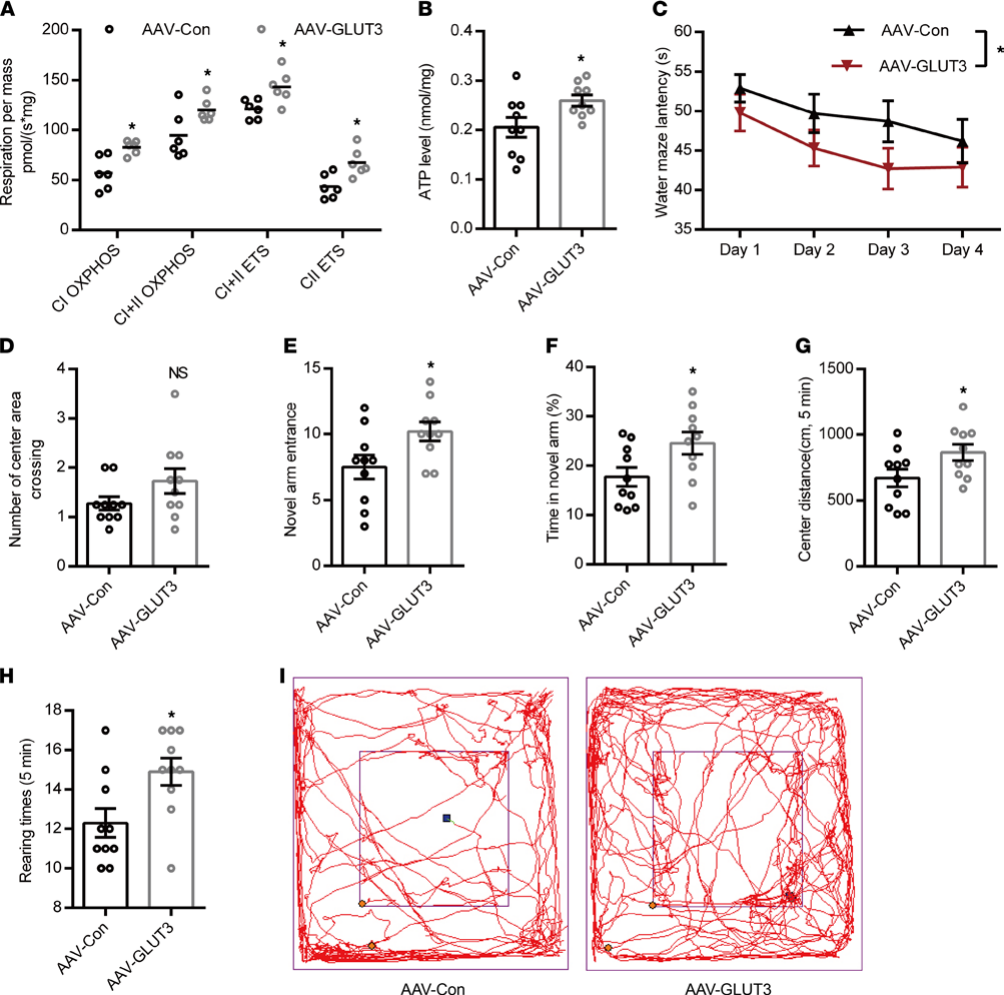
GLUT3 restoration improves mitochondrial dysfunction and cognitive deficits in APP/PS1-DM-RH mice. (**A**) High-resolution respirometry measured the oxygen consumption capacity of hippocampal mitochondria (*n* = 6 mice for each group). (**B**) ATP content of hippocampus (*n* = 9 mice for each group). (**C **and** D**) Morris water maze test. (**C**) Escape latency during platform trials and (**D**) number of center area crossing in probe test. (**E**) Novel arm entrance and (**F**) time spent in the Novel arm in Y-maze test. (**G**) Distance traveled in center region, (**H**) number of rearing, and (**I**) representative tracing graphs in the open-field test. *n* = 10 mice for each group. The data are expressed as the mean ± SEM. Statistical significance was assessed using 2-way ANOVA followed by Sidak’s multiple-comparison test in **A** and **C** and unpaired Student’s *t* test in **B** and **D**–**H**. *P < 0.05.

Then, an adeno-associated virus (AAV2/9) vector carrying mouse *slc2a3* gen, selectively transducing neurons, was injected into the hippocampal DG area of 4-month-old APP/PS1-DM mice (AAV-GLUT3) and the control group (AAV-Con) received the same dose of AAV vector. Three days after virus injection, these 2 groups of mice received RH treatment for 8 weeks. GLUT3 abundance in the DG area of AAV-GLUT3 mice (Supplemental Figure 8A) was significantly higher than that in AAV-Con mice. GLUT3 overexpression markedly reduced the levels of Aβ40 and Aβ42 (Supplemental Figure 8B) and levels of proinflammatory cytokines, including TNF-α, IFN-γ, IL-1β, and IL-6 in the DG area of the hippocampus in APP/PS1-DM-RH mice (Supplemental Figure 8C), indicating that GLUT3 overexpression ameliorates RH-induced AD-type pathology. Additionally, the mitochondrial respiratory function and ATP content were significantly increased by GLUT3 overexpression (Figure 6, A and B). More importantly, we compared performances in behavioral tests between AAV-Con mice and AAV-GLUT3 mice, and results showed that GLUT3-overexpressed APP/PS1-DM-RH mice performed better than control mice in the Morris water maze test (Figure 6, C and D), the Y-maze test (Figure 6, E and F) and the open-field test (Figure 6, G–I). Taken together, these findings demonstrate that RH accelerates the progression of AD-type pathologies and cognitive deficits in STZ-induced APP/PS1-DM mice by inhibiting GLUT3-mediated glucose uptake.

### TRPC6 activation enhances Glut3-mediated glucose uptake in the brain and alleviates RH-induced cognitive deficits.

**Figure 7:**
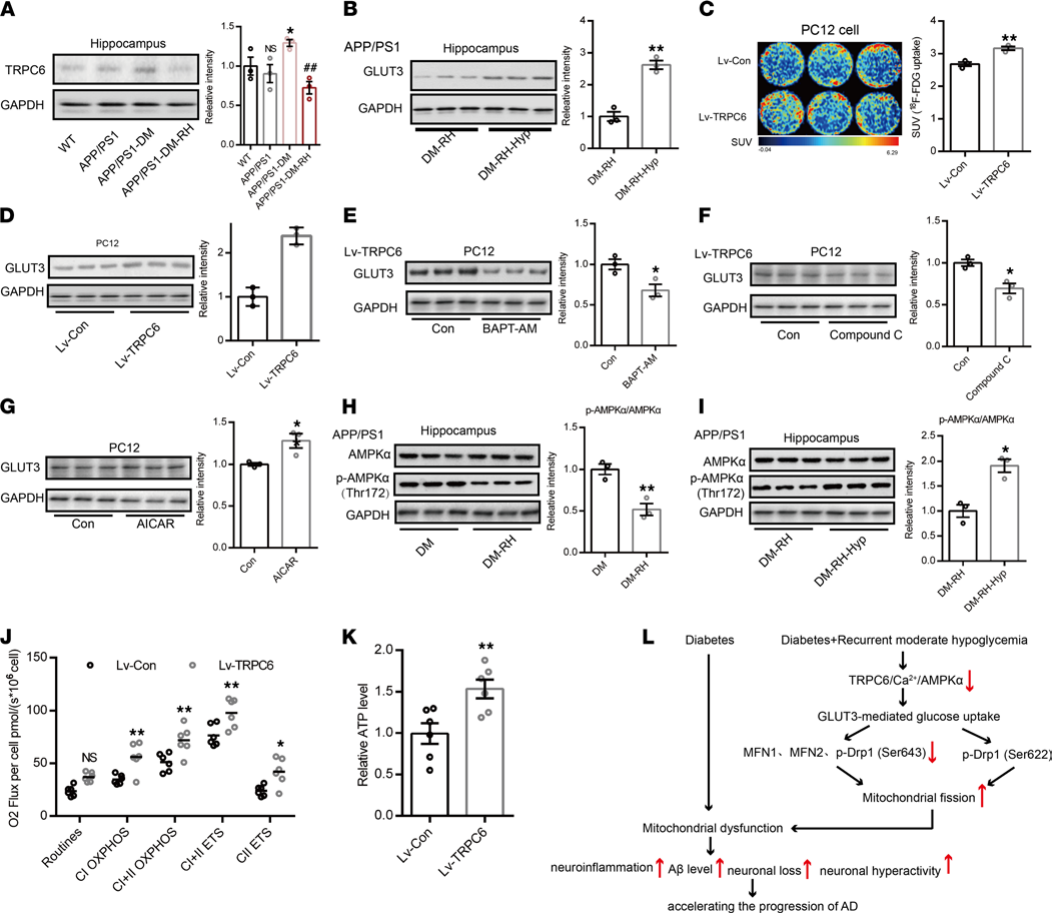
TRPC6 regulates GLUT3-mediated glucose uptake and mitochondrial function. (**A**) Western blot and quantitation for TPRC6 expression and (**B**) GLUT3 expression in hippocampus (*n* = 3 mice for each group). (**C**) Representative images for glucose uptake measured by ^18^F-FDG PET/CT scanning in PC12 cells. SUV of ^18^F-FDG is shown on right (*n* = 3 for each group). (**D**) Western blot and quantitation for GLUT3 expression in PC 12 cells with or without TRPC6 expression (*n* = 3 for each group). (**E **and** F**) Western blot and quantitation for GLUT3 expression in TRPC6-overexpressed PC 12 cells treated with BAPT-AM (**E**, 2 μM) or Compound C (**F**, 10 μM). (**G**) Western blot and quantitation for GLUT3 expression in PC 12 cells treated with AICAR (1 mM). (**H**) Western blot and quantitation for hippocampal AMPKα and p-AMPKα (Thr172) expression in APP/PS1-DM mice with or without RH treatment (*n* = 3 mice for each group). (**I**) Western blot and quantitation for hippocampal AMPKα and p-AMPKα (Thr172) expression in APP/PS1-DM-RH mice with or without hyperforin treatment (*n* = 3 mice for each group). (**J **and** K**) The mitochondrial respiratory function and ATP level in PC12 cells (*n* = 6 for each group). (**L**) A schematic diagram of this study. The data are expressed as the mean ± SEM. Statistical significance was assessed using unpaired Student’s *t* test in **A**–**I** and **K**, 2-way ANOVA followed by Sidak’s multiple-comparison test in **J**, or 1-way ANOVA. *P < 0.05, **P < 0.01; ^##^*P* < 0.01; NS, no significant difference.

To investigate whether GLUT3 expression was regulated by TRPC6, we first measured TRPC6 expression in the hippocampus. Western blotting show that TRPC6 expression was significantly increased in APP/PS1-DM mice but that RH significantly reduced TRPC6 expression (Figure 7A). There was no significant difference in TRPC6 expression between APP/PS1 and WT mice (Figure 7A). Immunofluorescent staining showed that TPRC6 expression was significantly reduced in the hippocampal CA1, CA3, and DG regions (Supplemental Figure 9, A–C). Long-term activation of TRPC6 with hyperforin, an agonist of TRPC6, significantly increased GLUT3 expression (Figure 7B) and brain glucose uptake (Supplemental Figure 10, A–L), along with mitochondrial function and ATP content in APP/PS1-DM-RH mice (Supplemental Figure 11, A and B). More importantly, hyperforin treatment significantly reduced Aβ deposition (Supplemental Figure 12A), GFAP expression (Supplemental Figure 12B), levels of IFN-γ, IL-1β, IL-6, and TNFα (Supplemental Figure 12C), and improved performance in behavioral tests (Supplemental Figure 13, A–G), suggesting reversal of cognitive deficits. Injecting AAV2/9-expressing mouse *TRPC6*-specific shRNA (shRNA-TRPC6) into the hippocampal DG area of APP/PS1 mice remarkably reduced the expression of TRPC6, p-AMPKα,and GLUT3, while the expression of GLUT1 and GLUT4 were not changed significantly (Supplemental Figure 14A). Importantly, the spatial memory and exploration ability in shRNA-TRPC6 mice were obviously impaired when compared with shRNA-Con mice (Supplemental Figure 14, B–H). In PC12 cells, lentivirus-mediated TRPC6 overexpression (Lv-TRPC6) led to higher ^18^F-FDG uptake and GLUT3 abundance compared with the control group (Lv-Con), indicating enhanced GLUT3-mediated glucose uptake (Figure 7, C and D). However, eliminating cytosolic Ca^2+^ with BAPT-AM or inhibiting AMPK activation using Compound C abolished the TRPC6 overexpression-induced increase in GLUT3 (Figure 7, E and F). Furthermore, activating AMPK with AICA Riboside (AICAR) significantly increased GLUT3 expression (Figure 7G), and while RH reduced the expression of p-AMPKα (Thr172) (Figure 7H), hyperforin increased it in the hippocampus (Figure 7I). Interestingly, the expression levels of Glut1, glut4, and SGLT1 were not altered significantly by TRPC6 overexpression (Supplemental Figure 15, A and B), and the expression of GLUT2, GLUT5, and SGLT2 were not detected in PC12 cells (Supplemental Figure 15, A and B). TRPC6 overexpression improved both mitochondrial function and ATP content in PC12 cells (Figure 7, J and K). Taken together, these results suggest that RH impairs Glut3-mediated glucose uptake by inhibiting the TRPC6/Ca^2+^/AMPK pathway, which not only leads to dysfunctional neuronal mitochondrial energy metabolism but also eventually accelerates the progression of cognitive deficits in STZ-induced APP/PS1-DM mice (Figure 7L).

## Discussion

Here, we demonstrate that 8 weeks of RH treatment dramatically aggravate cortical hyperactivity, promote the progression of cognitive deficits in APP/PS1-DM mice, and significantly impair GLUT3-mediated neuronal glucose uptake, which is associated with neuronal TRPC6 dysfunction. These data suggest that RH is a potent risk factor that can facilitate AD development and that TRPC6 might be a potential target for alleviating hypoglycemia-associated cognitive impairment. However, the conclusion in this study was drawn by using STZ-induced type 1 diabetic mice, not type 2 diabetic animal model; thus, these findings may not apply to patients with type 2 diabetes.

Diabetes mellitus is highly prevalent worldwide and is recognized as one of the major risk factors for cognitive decline and AD. Notably, up to half of all AD cases are potentially attributable to modifiable risk factors (e.g., diabetes, hypertension, and obesity) (35), and although various hypoglycemic drugs have been used to treat diabetes, glycemic control alone fails to prevent cognitive decline in patients with diabetes. Thus, it is critical to uncover how disturbances in glucose homeostasis lead to cognitive decline in patients with diabetes who are susceptible to AD. Recurrent hypoglycemia is a common complication in patients with diabetes, and several clinical trials have demonstrated that even 1 episode of severe hypoglycemia can significantly increase the risk of dementia in elderly patients with diabetes (10, 36, 37). As episodes of moderate hypoglycemia are more frequent and usually imperceptible in patients with diabetes, the detrimental effects of RH on the development of AD are frequently neglected. Here, we show that 6-month-old APP/PS1 mice had mild Aβ deposition, negligible neuronal loss and neuroinflammation, and normal cognitive performances. Diabetic APP/PS1 mice with insulin-controlled hyperglycemia had only slight cognitive deficits. However, 8 weeks of RH treatment dramatically accelerated the progression of AD-type pathologies and cognitive impairment in APP/PS1-DM mice, indicating that RH can promote AD progression. In nondiabetic APP/PS1 mice, the cognitive function was also impaired by 8 weeks of RH treatment, which indicates that RH-induced cognitive function under nondiabetic condition is directly caused by hypoglycemia rather than due to signals that respond to hypoglycemia. However, diabetes is a complex clinical syndrome and the cognitive deficits induced by RH may result from the glucose-independent effects under diabetic condition.

Neuronal hyperactivity is an early functional impairment in AD transgenic mice (38), wherein, while Aβ alone promotes hyperactivity, tau suppresses activity and promotes silencing of neurons (34). We show that hyperglycemia increased both neuronal silencing and hyperactivity in APP/PS1 mice but that RH only increased the fraction of hyperactive neurons in APP/PS1-DM mice; the latter observation is corroborated by greater Aβ deposition.

Normal neuronal activity is dependent on glucose homeostasis, and its disturbance is one of the major features of AD. Decreased brain glucose metabolism reflects synaptic activity deficit in the brain (39). However, whole brain glucose uptake was normal in 6-month-old APP/PS1 mice, which concurs with results from previous studies that have reported significant decline in brain glucose uptake in APP/PS1 mice but only at age 12 months. Importantly, our results indicate that while normal brain glucose uptake can be maintained despite underlying hyperglycemia, it is impaired by vigorous fluctuations in brain glucose, as seen during RH.

Glucose is transported across cell membranes by GLUTs and SGLTs and the human brain expresses several GLUT proteins and SGLT proteins. In patients with AD, the levels of GLUT1 and GLUT3, the major brain glucose transporters, are decreased in the cerebral cortex (40). In this study, we report that GLUT1 expression in the hippocampus of APP/PS1-DM mice is increased by RH, and several studies have revealed that insulin-induced hypoglycemia in diabetic rats increases GLUT1 expression in the BBB, which is essential for maintaining the glucose supply required for neurological functions (41, 42). Given the observed reduction in brain glucose uptake and activated neuroinflammatory response, we believe that increased GLUT1 expression is due to astrocyte activation, as reflected by enhanced glial fibrillary acidic protein (GFAP) expression, rather than as a compensatory response to hypoglycemia. One study has reported that hypoglycemia for 8 days increased neuronal GLUT3 expression, reflecting a neuron-specific adaptation against hypoglycemia (43). Here, our results showed that the GLUT3 expression and standard uptake value of FDG were significantly reduced by 8 weeks of RH treatment in APP/PS1 mice and APP/PS1-DM mice. We believe that the contradiction of GLUT3 expression and glucose uptake may have resulted from the intensity of intervention during 8 weeks of RH treatment, which disturbs the adaption caused by 8 days of provocation. It has also been demonstrated that diabetes causes severe cerebrovascular injury, leading to reduced cerebral blood flow and glucose supply to the brain (44–46). In this study, hippocampal GLUT3 expression in APP/PS1-DM mice were significantly higher than that of APP/PS1 mice, even though glucose uptake remained unchanged, indicating that the increase in GLUT3 expression is an adaptive response to diabetes to maintain adequate neuronal glucose supply; however, RH disturbs this adaptation in APP/PS1-DM mice. Thus, even though GLUT3 expression in APP/PS1-DM-RH mice was comparable to that of WT and APP/PS1 mice, glucose uptake was significantly reduced. Additionally, we cannot rule out the possibility that the impaired glucose uptake resulted from neuroinflammation and neuronal loss caused by RH.

Transporter-mediated glucose delivery to neurons is predominantly diverted to the mitochondria for ATP production; thus, its recurrent shortage may trigger mitochondrial dysfunction and cause brain energy stress. Indeed, ATP production from glucose metabolism declines dramatically in late-onset sporadic AD, and this tendency continues throughout the progression of the disease (47, 48). We show that RH induced hippocampal mitochondrial fragmentation, which was substantiated by the reduction in MFN1, MFN2, and p-Drp1 (Ser 643) abundance, along with cognate reduction in oxidative phosphorylation and ATP content. GLUT3 overexpression significantly enhanced glucose uptake, mitochondrial function, and ATP content in PC12 cells. Thus, excessive hippocampal mitochondrial fission caused by RH could be a response to the glucose shortage caused by impairment of GLUT3-mediated glucose uptake in APP/PS1-DM mice. However, the mechanisms underlying changes in MFN1/2 expression and Drp1 activity require further investigation.

We have recently reported that TRPC6 is a critical sensitive cation channel to hypoglycemia and a promising target for preventing RH-induced cognitive impairment (26). Here, we extend these observations and link the TRPC6/Ca^2+^/AMPK pathway and GLUT3 expression in APP/PS1-DM-RH mice, i.e., that RH reduces hippocampal TRPC6/AMPKα/GLUT3 expression but that hyperforin-induced long-term activation of TRPC6 reverses this effect and delays the onset of severe cognitive impairment. Similar results were seen with PC12 cells, as GLUT3 expression was significantly inhibited by BAPT-AM and Compound C but increased upon AMPK activation by AICAR. We recently showed that the GLP-1 receptor agonist, liraglutide, can improve the cognitive function of patients with type 2 diabetes mellitus (49). Thus, the protective effects of hyperforin on cognitive function should be further tested in patients with diabetes. Taken together, we show that hyperglycemia only slightly impairs cognitive function but that RH significantly promotes the progression of AD by inhibiting TRPC6/GLUT3-mediated glucose uptake in APP/PS1-DM mice. Activation of TRPC6 with hyperforin could delay the present of dementia caused by RH. Therefore, we propose that hypoglycemic treatment in patients with diabetes with AD risk should adopt the “better high than low” strategy to avoid recurrent episodes of moderate hypoglycemia.

## Methods

### STZ-induced APP/PS1-DM mice.

APP/PS1 transgenic mice and WT mice (C57BL/6) were purchased from Junke Biological and bred in the animal room of the institute. To establish the diabetic model, streptozotocin (50 mg/kg/day) was injected i.p. for 5 consecutive days in 4-month-old male APP/PS1 mice. At 3 days after the final injection, mice with RBG ≥ 16.7 mM were defined as STZ-induced APP/PS1-DM mice. To ensure animal health and to replicate blood glucose control in patients with diabetes, diabetic mice were administrated insulin (glargine) once a day. The starting dose of 3 IU/kg was injected subcutaneously, and the dose was adjusted according to the glucose levels. All procedures followed guidelines issued by the University Animal Welfare Committee.

### RH and hyperforin treatment.

Hypoglycemia (2.2–3.9 mM) was induced in 4-month-old mice in the APP/PS1-DM group by injecting regular insulin (hypodermic injection [i.h.], 5.0 units/kg, started at 15:00) thrice weekly. This was continued for a period of 8 weeks, i.e., until the animals were 6 months old (APP/PS1-DM-RH). Blood glucose level was monitored using a glucometer, each hypoglycemia episode was maintained for about 2 hours, and mice were subsequently provided free access to food to restore glycemic status. If blood glucose was lower than 2.2 mM, glucose (1 g, intragastric) was immediately administered to avoid severe hypoglycemia. Mice that suffered severe hypoglycemia were excluded from the study. Some RH-treated mice were administrated hyperforin (6 mg/kg, i.p.; Sigma, PHL89225) for 8 weeks (APP/PS1-DM-RH-Hyp). All the mice in the control group were similarly treated with saline. One week after completion of the RH treatment, all animals underwent behavioral tests followed by ^18^F-FDG PET/CT and 2-photon imaging. All these procedures were conducted under euglycemic conditions.

### ^18^F-FDG PET/CT imaging.

Glucose uptake in the brain and in PC12 cells was assessed on a Micro-PET-CT scanner (Pingseng Healthcare). On the day of experiment, STZ-induced APP/PS1-DM mice were not treated with insulin. Specifically, mice were anesthetized with isoflurane and injected with ^18^F-FDG (approximately 8 MBq, i.v.) after fasting for 12 hours. PC12 cells were seeded in 6-well plates and incubated with ^18^F-FDG (10 μCi per well) in low-glucose medium at 37°C for 45 minutes. PET images, 10 minute dynamic 3-dimensional scans with an energy window of 350–650 keV, were acquired and CT was used to obtain anatomical reference images. Uptake rates of ^18^F-FDG were analyzed as volumes of interest (VOIs) were drawn over the entire brain. The rate of glucose utilization was measured using SUVs (SUV = [^18^F-FDG activity in each VOI (Bq / mL)] / [injected dose in Bq] / [body weight (g)]).

### Behavioral tests.

Animals underwent behavioral testing, namely, the Morris water maze, the Y-maze, and open-field testing, for evaluation of spatial learning, memory, and exploration ability. The mice were brought to the test room for adaptation 3 days prior to the test. In the open-field test, mice were placed in the center of the apparatus and allowed free exploration for 5 minutes. Paths were recorded and parameters, such as total distance traveled and distance to central area, were measured. In the Y-maze test, mice were allowed to move freely in 2 arms (home arm and familiar arm) with the other arm (novel arm) blocked for 3 minutes. After a 2 hour interval, mice were allowed to freely explore all 3 arms for 5 minutes, and the number of novel arm entries and time spent in the novel arm were recorded. The Morris water maze test was conducted on a trial platform and comprised 4 platform tests per day for 4 consecutive days with a probe trial on the 5^th^ day. Swimming capabilities of the animals were evaluated before the test.

### In vivo 2-photon Ca^+2^ imaging.

All procedures were performed as described in a previous study (50). Briefly, animals were anesthetized by inhalation of 1.5%. After removing the skin, a customized recording chamber with a hole at the front was cemented to the skull with cyanoacrylic glue (UHU). Next, a small craniotomy (1.5 × 1.5 mm) was created at the projecting point of the frontal cortex (1.5 mm lateral to the middle and 2.9 mm anterior to the bregma), bleeding was stopped, and 1.5% agarose was layered on the exposed cortex to suppress pulsation. Respiration rate was maintained between 90 and 110/min. Neurons in layer 2/3 of the prefrontal cortex were bulk loaded with 0.5 mM Ca^2+^ indicator Cal-520 AM (AAT-Bioquest) under a 2-photon microscope. Imaging was performed on a 2-photon microscope with a 12-kHz resonant scanner (model LotosScan 1.0, Suzhou Institute of Biomedical Engineering and Technology). A laser source provided excitation light (λ = 920 nm; Coherent) through a water-immersion objective (Nikon, 40X, NA 0.8) and a consecutive recording (4–6 mins) was acquired at a 40-Hz frame rate using custom-written software based on LabVIEW (National Instruments). Data analysis was performed using LabVIEW 2014 (National Instruments) and Igor Pro 5.0 (Wavemetrics). Glial cells were excluded based on morphology and time course of Ca^2+^ transients.

### Stereotactic injection.

AAV2/9 vectors carrying shRNA targeting mouse *Trpc6* (AAV2/9-H1-shRNA-CAG-EGFP-WPRE-pA, shRNA-TRPC6; Taitool Biotechnology) or mouse *slc2a3* gen (AAV2/9-hSyn-EGFP-P2A-slc2a3-3xflag-WPRE, AAV-GLUT3) and control virus (shRNA-Con, AAV-Con) were bilaterally injected into the hippocampus DG region of APP/PS1 mice or APP/PS1-DM mice, respectively. Specifically, the mice were fixed in the stereotaxic apparatus and anesthetized with isoflurane. Then, a small craniotomy was created at the projecting point of the DG region (AP = −1.9; ML = ± 1.1; DV = −2.0). Virus was injected at a speed of 0.1 μL/min with Hamilton needle by using an automatic microinjection system (World Precision Instruments) and the needle was left in the injection point for 10 minutes before being slowly retracted. Three days after injection, mice from the AAV-Con and AAV-GLUT3 groups received RH treatment. Four weeks after injection, mice from the shRNA-Con and shRNA-TRPC6 groups underwent behavioral tests.

### Histopathological staining.

Free-floating 12 mm serial coronal sections of the brain were cut on a freezing microtome (Leica) and washed 3 times. For immunofluorescent staining, sections were blocked with Immunol Staining Blocking Buffer and incubated with primary antibody (anti-NeuN; Abcam, ab177487), anti-MAP-2 (Abcam, ab5392), anti-GFAP (Abcam, ab7260), anti-GLUT3 (Alomone Lab, GTX129175), and anti-TRPC6 (Millipore, T6442) overnight at 4°C. After washing, sections were incubated with secondary antibody diluted in Secondary Antibody Dilution Buffer (Beyotime) for 45 minutes at 37°C. Anti-beta amyloid antibody (Abcam, ab2539) and anti-Iba1 antibody (Abcam, ab178847) was used for beta amyloid and Iba1 visualization. Images were acquired on a Leica laser confocal microscope or an optical microscope, and the area fraction of positive staining against the area of tissue analyzed in the neocortex and the hippocampus were quantified using ImageJ (NIH) software.

### ELISA.

Animals were sacrificed, the brains were quickly harvested, and the hippocampus homogenized in liquid nitrogen. Concentrations of Aβ40, Aβ42 (R&D Systems, DAB140B, DAB142), HbA1c (MSK, kt20296), IL-6, IL-1β, INF-γ, and TNF-α (Boster, EK0411, EK0394, EK0375, and EK0527, respectively) in tissue homogenate were quantitatively measured by ELISA according to manufacturers’ instructions.

### Mitochondrial morphology, function, and ATP content.

Hippocampal morphology was assessed using TEM, and approximately 100 mitochondria per sample were used for morphometry. Mitochondria from tissue or cells were extracted using the Mitochondria Isolation Kit (Beyotime) and high-resolution respirometry (O2K) was used to measure respiratory function, as previously described (26). ATP content was determined using the Enhanced ATP Assay Kit (Beyotime).

### Cell culture and lentiviral infection.

Well-differentiated PC12 cells were purchased from Procell and cultured in DMEM medium supplemented with 10% FBS and 1% streptomycin/penicillin. Recombinant Lv vector (Ubi-MCS-3FLAG-SV40-EGFP-IRES-puromycin) purchased from GENECHEM was used for TRPC6 or GLUT3 overexpression. To evaluate the role of Ca^2+^ signaling, BAPT-AM (2μM, Ca^2+^ chelator), Compound C (10 μM, MCE), or AICAR (1 mM, MCE) was added.

### Western blot.

Hippocampal tissues or cells were homogenized in buffer (0.5 mol/L Tris; 1% NP40; 1% Triton X-100; 1 g/L sodium dodecyl sulfate; 1.5 mol/L NaCl; 0.2 mol/L EDTA; 0.01 mol/L EGTA; and protease inhibitor and/or phosphatase inhibitor), sonicated, and incubated at −20°C for 20 minutes, followed by centrifugation at 12,000 *g* for 20 minutes at 4°C. The supernatant was collected and protein concentration determined by the bicinchoninic acid (BCA) method. Next, 50 μg protein were loaded on 10% SDS polyacrylamide gel. The primary antibodies used were anti-TRPC6 (Alomone, ACC-017), anti-PSD 95 (Abcam, ab238135), anti-SNAP25(Abcam, ab109105), anti-SYP (Abcam, ab32127), anti-MFN1 (Abcam, ab126575), anti-MFN2 (Abcam, ab124773), anti-Drp1(Abcam, ab184247), anti-p-Drp1(mice Ser 622; CST, 3455), anti-p-Drp1 (mice Ser 643; Abcam, ab193216), anti-GLUT1-5 antibody (Affinity, AF6731, DF7510, AF5463, AF5386, DF13545), anti-SGLT1 (Invitrogen, PA5-77460), and anti-SGLT2 (Abcam, ab137207), followed by incubation with the secondary antibodies (ZSGB-BIO). Protein expression was normalized to GAPDH intensity or total protein content. See complete unedited blots in the supplemental material.

### Statistics.

Data are expressed as mean ± SEM. Statistical differences were assessed using the 2-tailed Student’s *t* test or 1-way or 2-way ANOVA with Bonferroni’s multiple-comparison post hoc tests, as appropriate. All analyses were conducted on SPSS 17.0, or GraphPad Prism software, version 6.0 (GraphPad Software). Two-sided *P* values less than 0.05 were regarded as statistically significant.

### Study approval.

Procedures were carried out with the approval of, and in accordance with, the Animal Ethics Committee at the Army Medical University, Chongqing.

## Author contributions

ZZ initiated the project. ZZ and CH designed the experiments. ZZ and CH wrote the paper. QZ and PG contributed to the experiments. CH, YZ, QL, LL, and YC collected and provided the data. CH, HM, LW, WS, and ZL analyzed the data. XC, HJ, GY, MT, HZ, DL, and ZZ critically read and revised the paper. All authors read and approved the manuscript.

## Supplementary Material

Supplemental data

## Version 1 01/25/2022

In-Press Preview

## Version 2 03/08/2022

Electronic publication
