# Supplementary material for: Recurrent moderate hypoglycemia accelerates the progression of Alzheimer’s disease through impairment of the TRPC6/GLUT3 pathway
Source: JCI Insight. 2022 Mar 8;7(5):e154595. doi: 10.1172/jci.insight.154595 (PMC8983129; doi:10.1172/jci.insight.154595)

Supplementary figures and legends

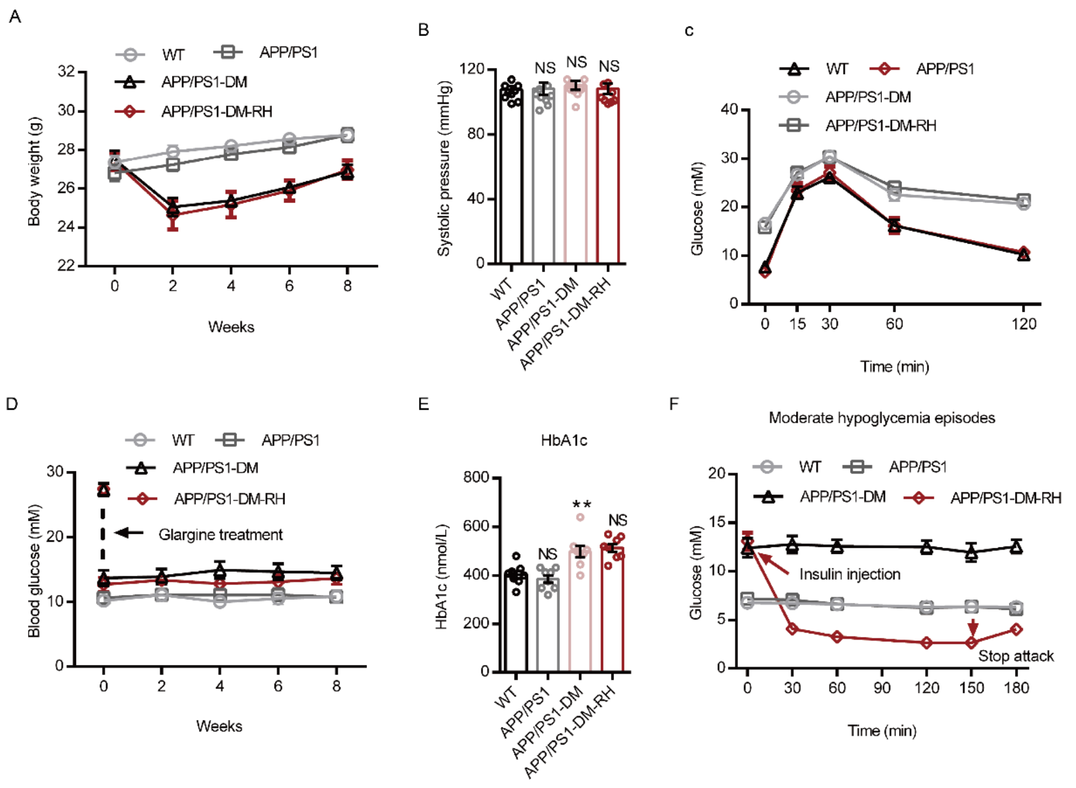

Figure S1, Related to Figure 1. The metabolic effects of RH. (A) Changes of body weight (n=10). **(B)** Blood pressure after completing RH treatment (n=10). **(C)** Intraperitoneal Glucose Tolerance Test (IPGTT) in mice after completing RH treatment (n=6). **(D)** The random blood glucose among 8 weeks of RH treatment (n=10). Glargine treatment, diabetic mice received glargine once a day. **(E)** The HbA1c in serum of mice (n=8 for each group). **(F)** Changes of blood glucose during a moderate hypoglycemia episode induced by insulin injection (n=10).The data are expressed as the mean ± SEM. Statistical significance was assessed using a one-way ANOVA (panel B and E) or two-way ANOVA (panel A, C and D) followed by Dunnett's multiple comparisons test. $^{***}P < 0.001$, APP/PS1-DM VS APP/PS1; WT VS APP/PS1, APP/PS1-DM-RH VS APP/PS1-DM, no significant difference (NS).

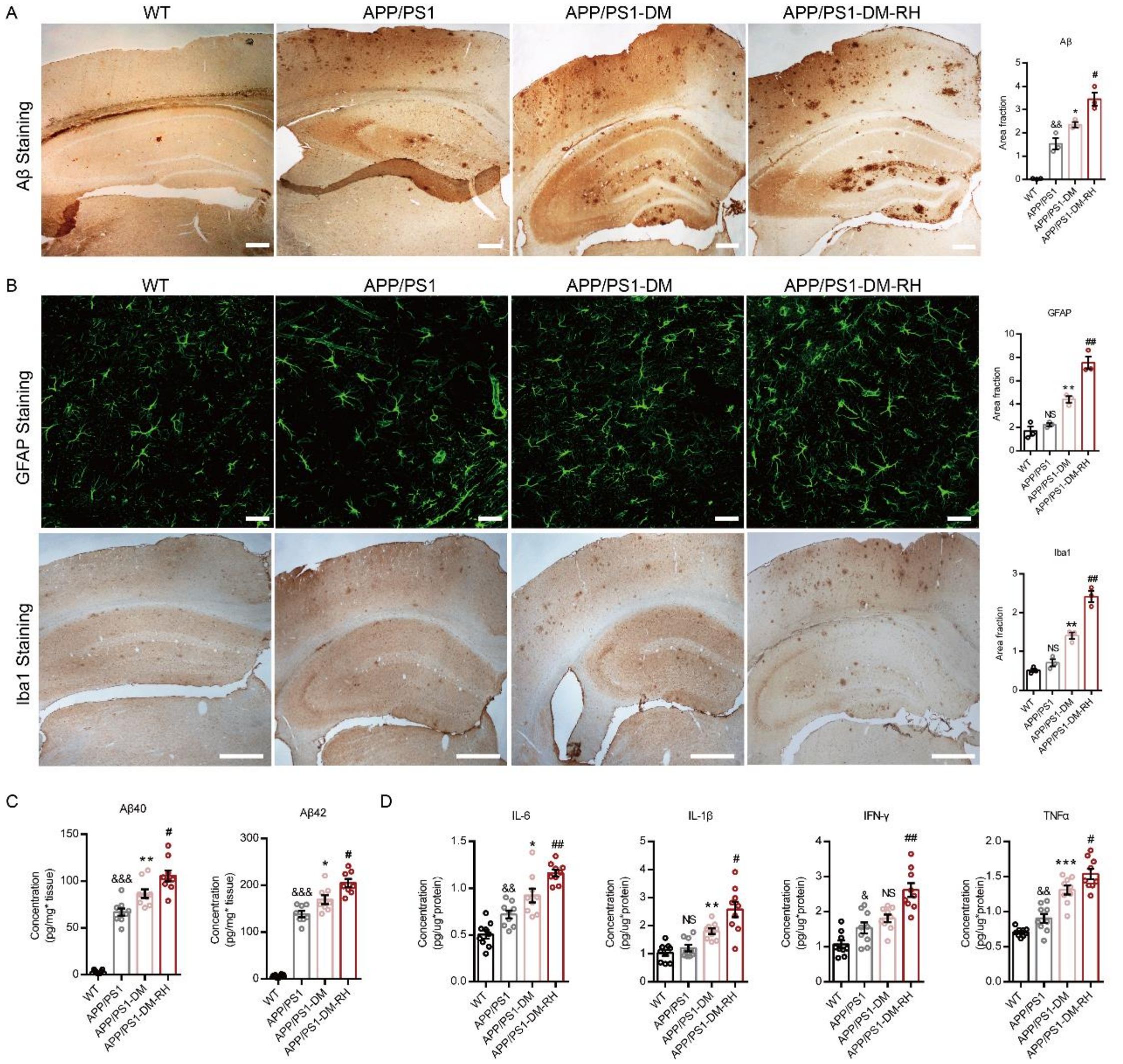

Figure S2, Related to Figure 2. AD-type pathology in hippocampus. (A) Immunohistochemical staining for Aβ deposition in hippocampus. Quantitation is shown on the right (n=3 mice for each group). **(B)** Immunofluorescent staining for GFAP (astrocyte marker, *top*) and immunohistochemical staining for Iba1 (microglia marker, *bottom*). Quantitation is shown on the right (n=3 mice for each group). **(C)** ELISA of Aβ40 and Aβ42 concentration in hippocampal homogenates (n=9 mice for each group). **(D)** Quantification of IL-6, IL-1β, IFN-γ and TNF-α by ELISA in hippocampal homogenates (n=9 mice for each group). The data are expressed as the mean ± SEM. Statistical significance was assessed using unpaired student's T test (panel A-C) one-way ANOVA followed by Dunnett's multiple comparisons test (panel D). $^{\&}P<0.05$ and $^{\&\&}P<0.05$, WT VS APP/PS1; $^{*}P<0.05$, $^{**}P<0.01$ and $^{***}P<0.01$, APP/PS1-DM VS APP/PS1; $^{\#}P<0.05$ and $^{\#\#}P<0.01$, APP/PS1-DM-RH VS APP/PS1-DM; NS, no significant difference.

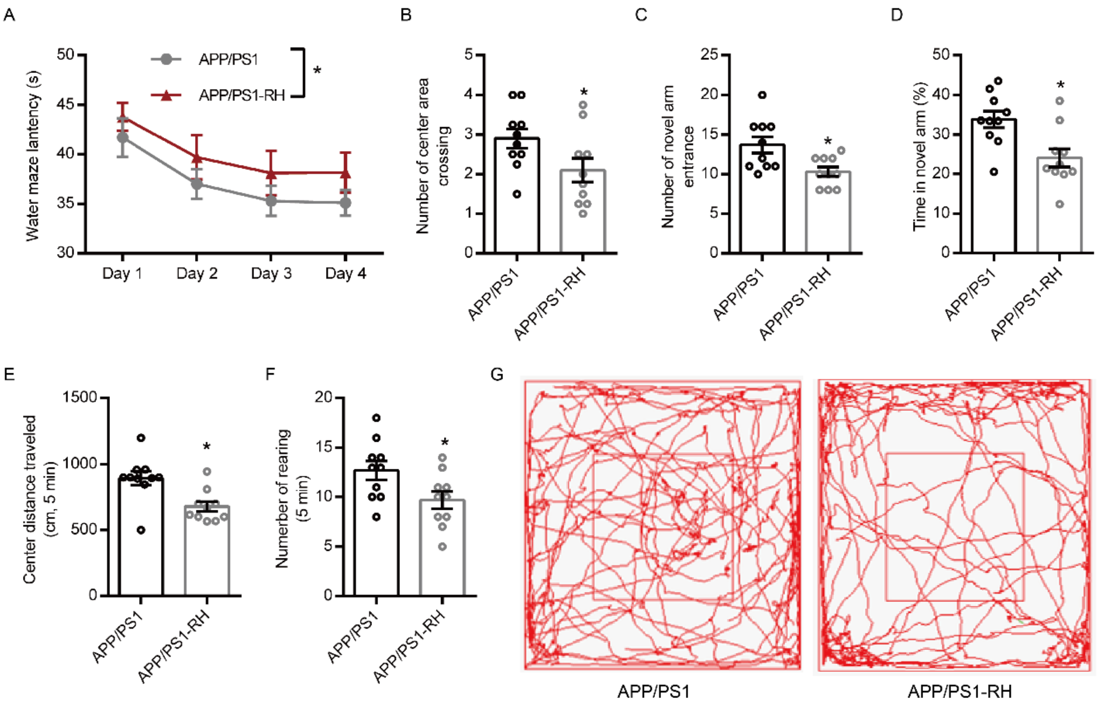

Figure S3, Related to Figure 3. RH impaired the performances of non-diabetic APP/PS1 mice in behavioral tests. (A and B) Morris water-maze test. Escape latency during platform trials (A) and number of center area crossing (B) in probe test. APP/PS1-RH, APP/PS1 mice received 8 weeks of RH treatment. **(C and D)** Novel arm entrance (C) and time spend in the novel arm (D) in Y-maze test. **(E-G)** Distance traveled in center area (E), number of rearing (F), and representative tracing graphs in open field test (G). n=10 for each group. The data are expressed as the mean ± SEM. Statistical significances were assessed using two-way ANOVA (panel A) or unpaired student's T test (panel B-F). $^{*}P<0.05$, $^{**}P<0.01$ versus APP/PS1.

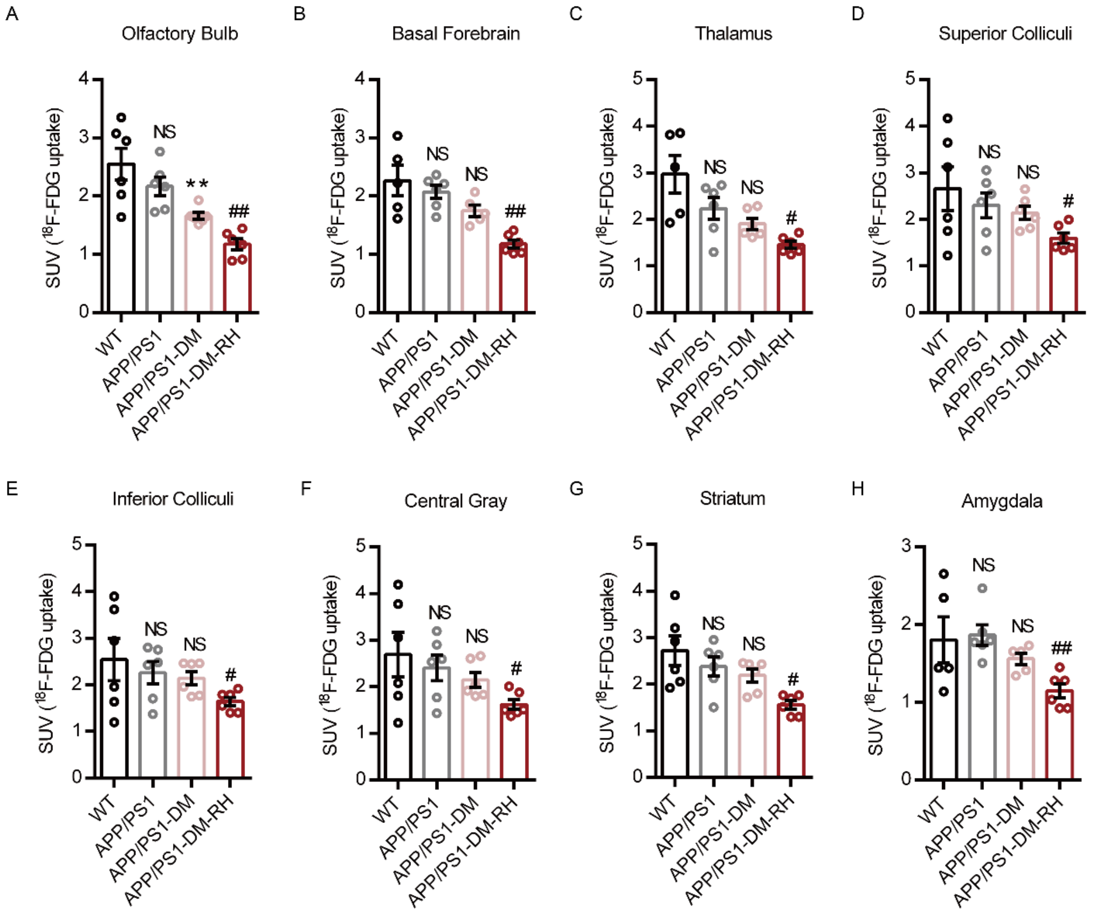

Figure S4, Related to Figure 5. ^{18}F-FDG uptake in brain. (A-H) 18F-FDG uptake in different brain regions measured by PET/CT scanning (n=6 mice for each group). The data are expressed as the mean ± SEM. Statistical significance was assessed using unpaired student's T test. $^{**}P<0.01$, APP/PS1-DM VS APP/PS1; $^{\#}P<0.05$ and $^{\#\#}P<0.01$, APP/PS1-DM-RH VS APP/PS1-DM; WT VS APP/PS1, no significant difference (NS).

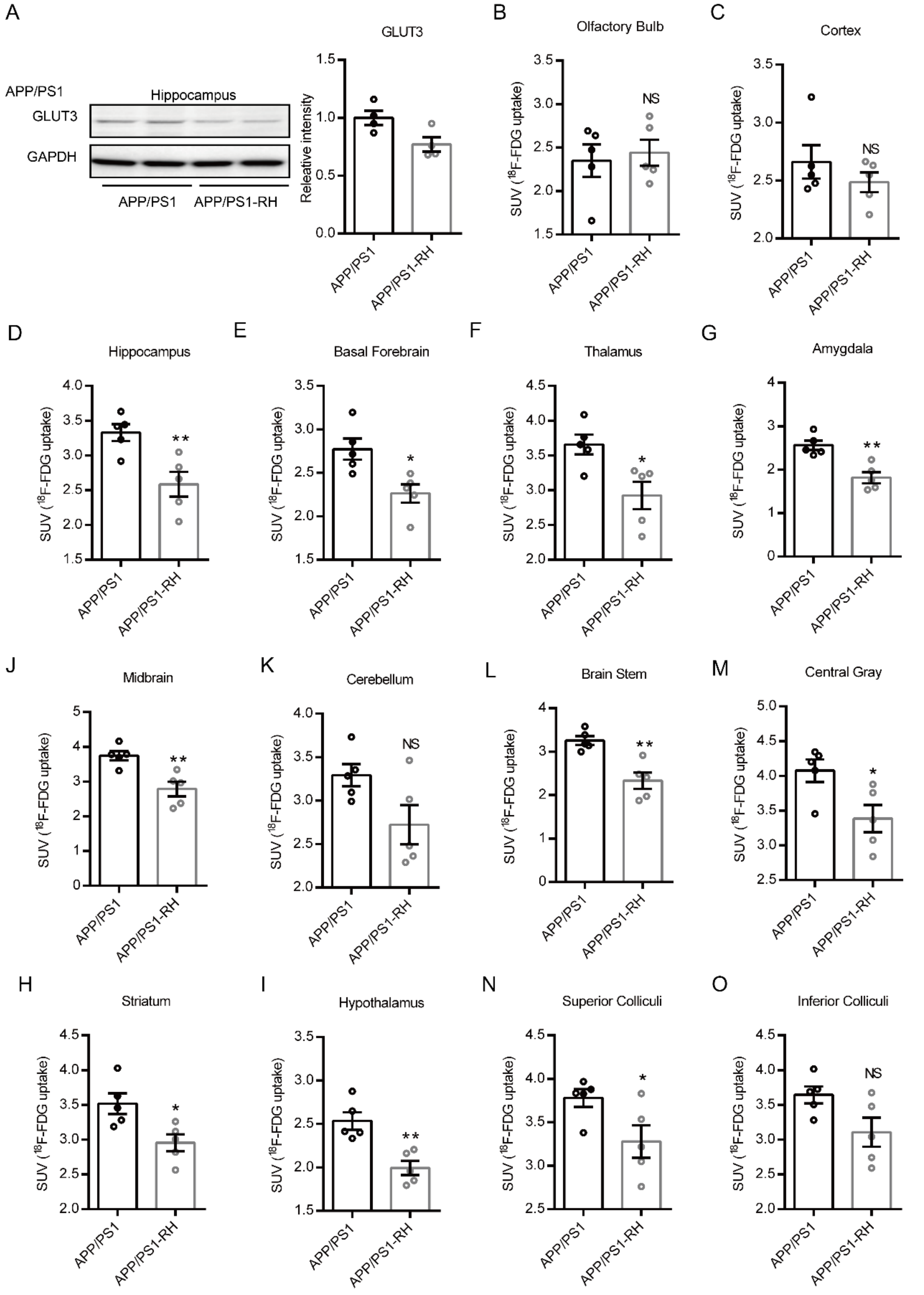

Figure S5, Related to Figure 5. RH reduced hippocampal GLUT3 expression and brain ^{18}F-FDG uptake in non-diabetic APP/PS1 mice. (A) Western-blotting and quantitative data shown the expression of hippocampal GLUT3 (n=4 mice for each group). APP/PS1-RH, APP/PS1 mice received 8 weeks of RH treatment. **(B-O)** The standard uptake value (SUV) of ^{18}F-FDG in brain area (n=5 mice for each group). The data are expressed as the mean ± SEM. Statistical significance was assessed using unpaired student's T test. *$P < 0.05$,**$P < 0.01$; NS, no significant difference (NS).

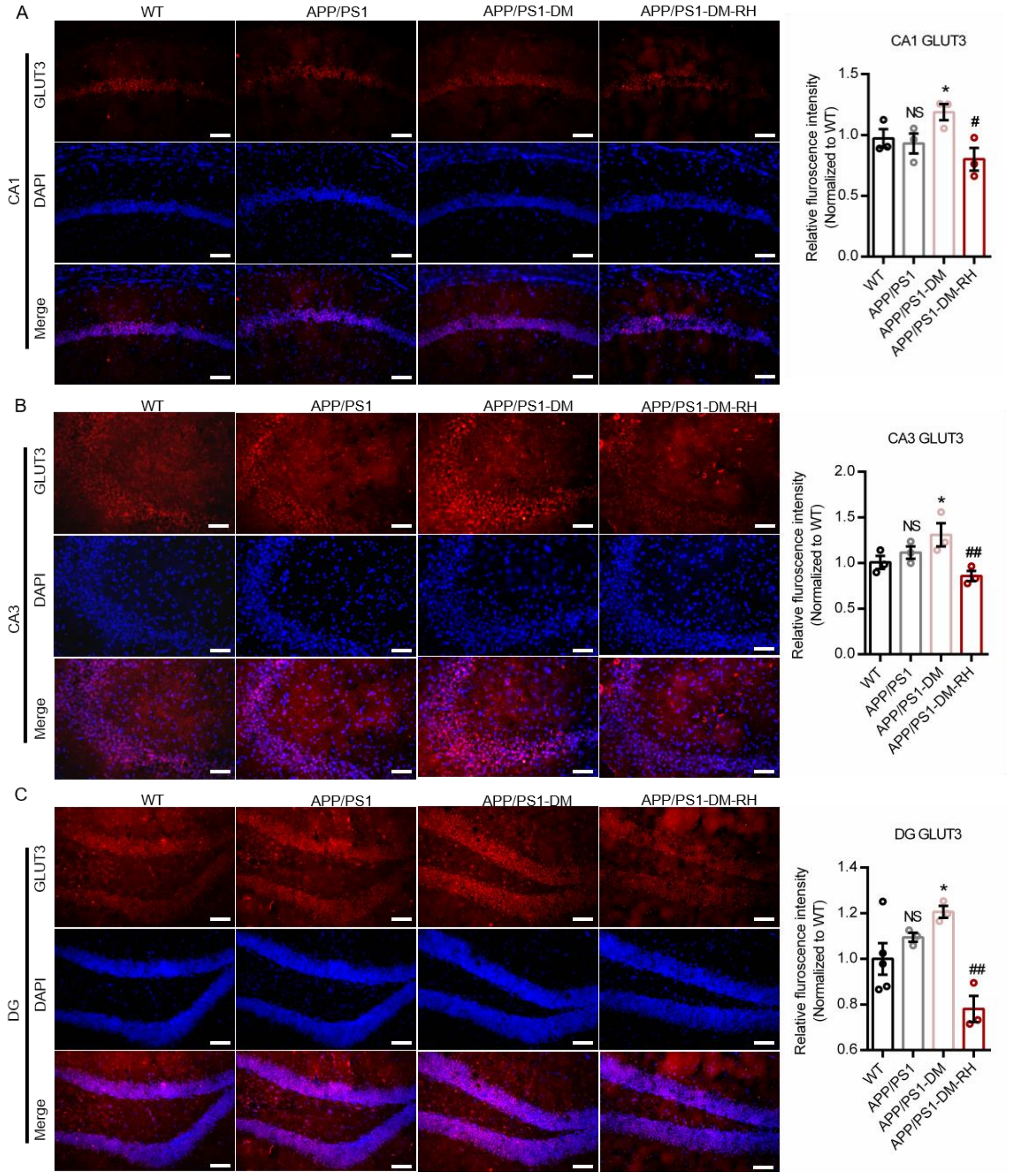

Figure S6, related to Figure 6. GLUT3 expression in hippocampus. (A-C) Representative images of GLUT3 (red) and DAPI (blue) immunostaining in hippocampal area of CA1 (A), CA3 (B) and DG (C) from WT, APP/PS1, APP/PS1-DM, and APP/PS1-DM-RH mice. Scale bar, 100μm. Quantitative results are showed in the right (n=3 mice for each group). The data are expressed as the mean ± SEM. Statistical significance was assessed using unpaired student's T test $^{*}P<0.05$, APP/PS1-DM VS APP/PS1; $^{\#}P<0.05$, $^{\#\#}P<0.01$ and $^{\#\#\#}P<0.001$, APP/PS1-DM VS APP/PS1-DM-RH; WT VS APP/PS1, no significant difference (NS).

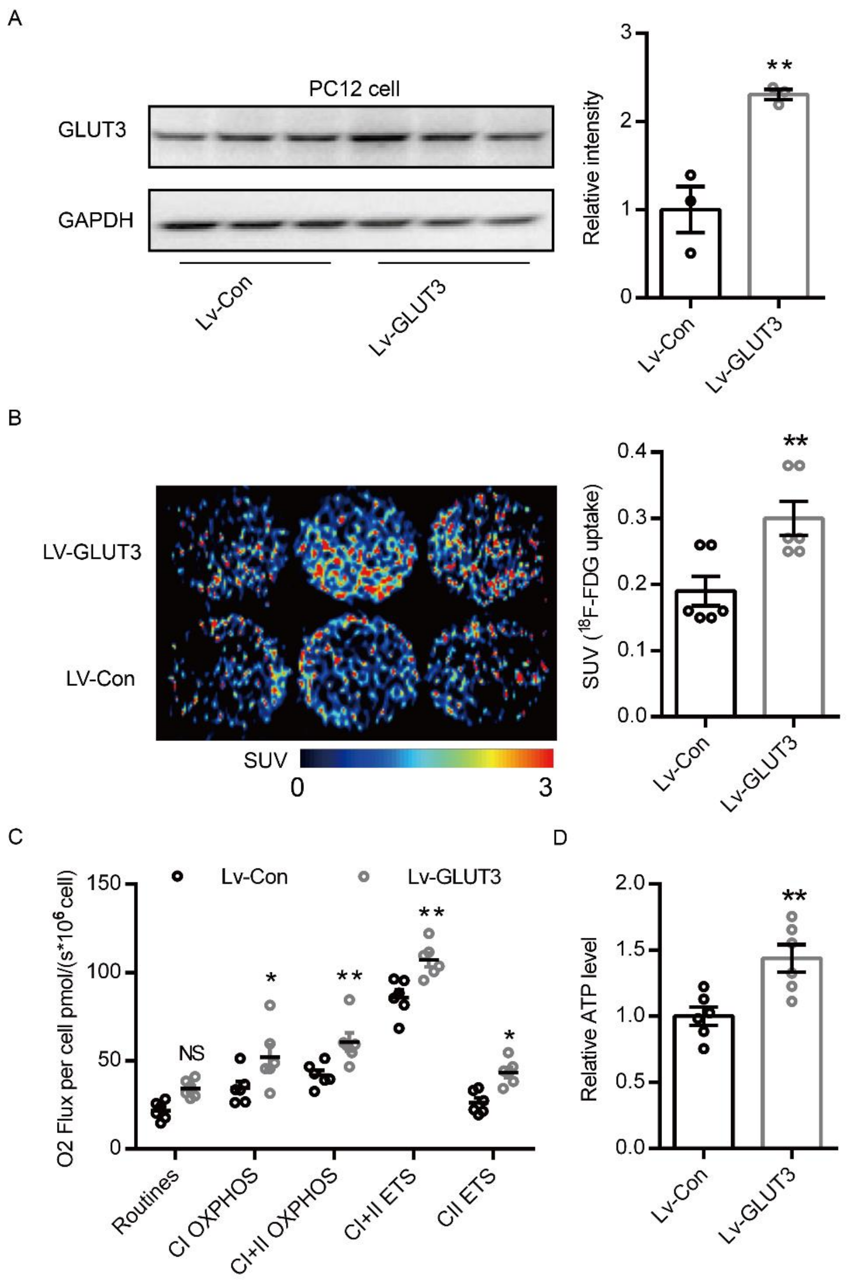

Figure S7, Related to Figure 6. GLUT3 over-expression in PC12 improves mitochondrial function in PC12 cells. (A) Western blotting to assess GLUT3 overexpression in PC12 cells. Lv-Con, PC12 cells infected with empty recombinant lentiviral vector; Lv-GLUT3, PC12 cells infected with GLUT3 recombinant lentiviral vector. (**B)** Representative image and quantitative results show the ^{18}F-FDG uptake in PC12 cells with or without GLUT3 overexpression (n=6 for each group). **(C)** High-resolution respirometry measured the oxygen consumption capacity of mitochondria in PC12 cells (n=6 for each group). **(D)** ATP content in PC12 cells (n=6 for each group). The data are expressed as the mean ± SEM. Statistical significance was assessed using unpaired student's t test (panel A,B and D) or two-way ANOVA followed by Sidak's multiple comparisons test (panel C). $*P<0.05$, $**P<0.01$.

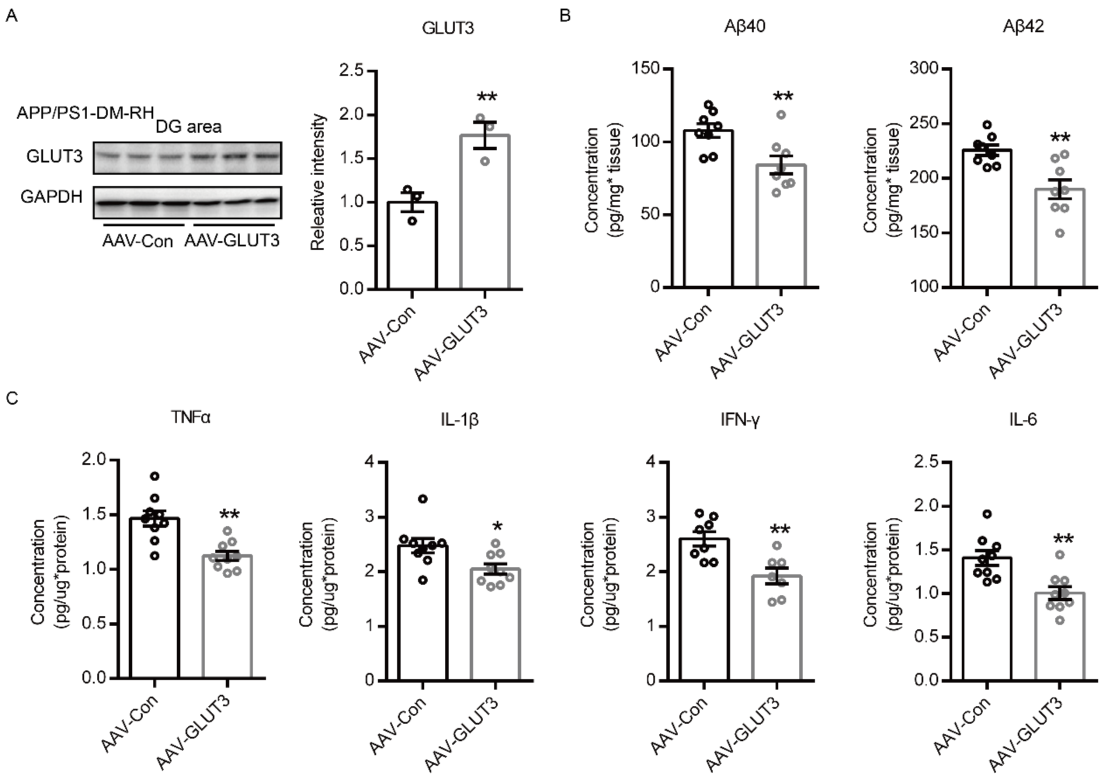

Figure S8, Related to Figure 6. GLUT3 over-expression in DG area of hippocampus improve RH induced AD-type pathologies in APP/PS1-DM-mice. (A) Western blot to assess GLUT3 abundance in DG area of hippocampus and the quantitative data shown in the right (n=3 mice for each group). AAV-Con, APP/PS1-DM-RH mice injected with empty adeno-associated virus in DG area; AAV-GLUT3, APP/PS1-DM-RH mice injected with adeno-associated virus expressing GLUT3 protein in DG area. (**B)** ELSIA to detect Aβ40 and Aβ42 in DG area of hippocampus (n=8 mice for each group). **(C)** Quantification of IL-6, IL-1β, IFN-γ and TNF-α by ELISA in DG area of hippocampus (n=9 mice for each group). The data are expressed as the mean ± SEM. Statistical significance was assessed using unpaired student's t test. *$P<0.05$, **$P<0.01$.

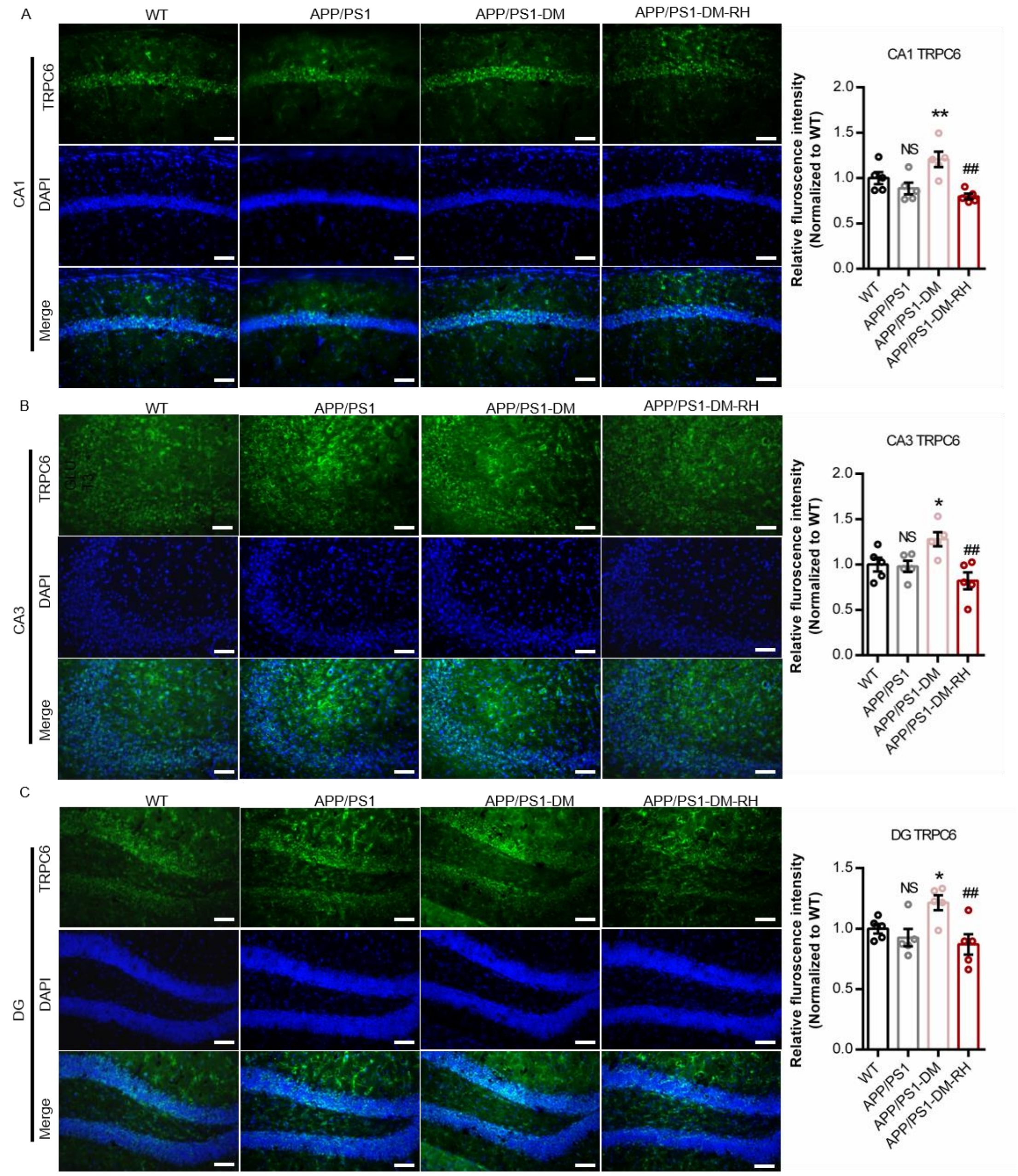

Figure S9, related to Figure 7. TRPC6 expression in hippocampus. (A-C) Representative images of TRPC6 (green) and DAPI (blue) immunostaining in hippocampal area of CA1 (A), CA3 (B) and DG (C) from WT, APP/PS1, APP/PS1-DM, and APP/PS1-DM-RH mice. *Scale bar*, 100μm. Quantitative results are showed in the right (n=5 mice for each group). The data are expressed as the mean ± SEM. Statistical significance was assessed using unpaired student's t test. $^{*}P < 0.05$, APP/PS1-DM VS APP/PS1; $^{\#\#}P < 0.01$, APP/PS1-DM VS APP/PS1-DM-RH; WT VS APP/PS1, no significant difference (NS).

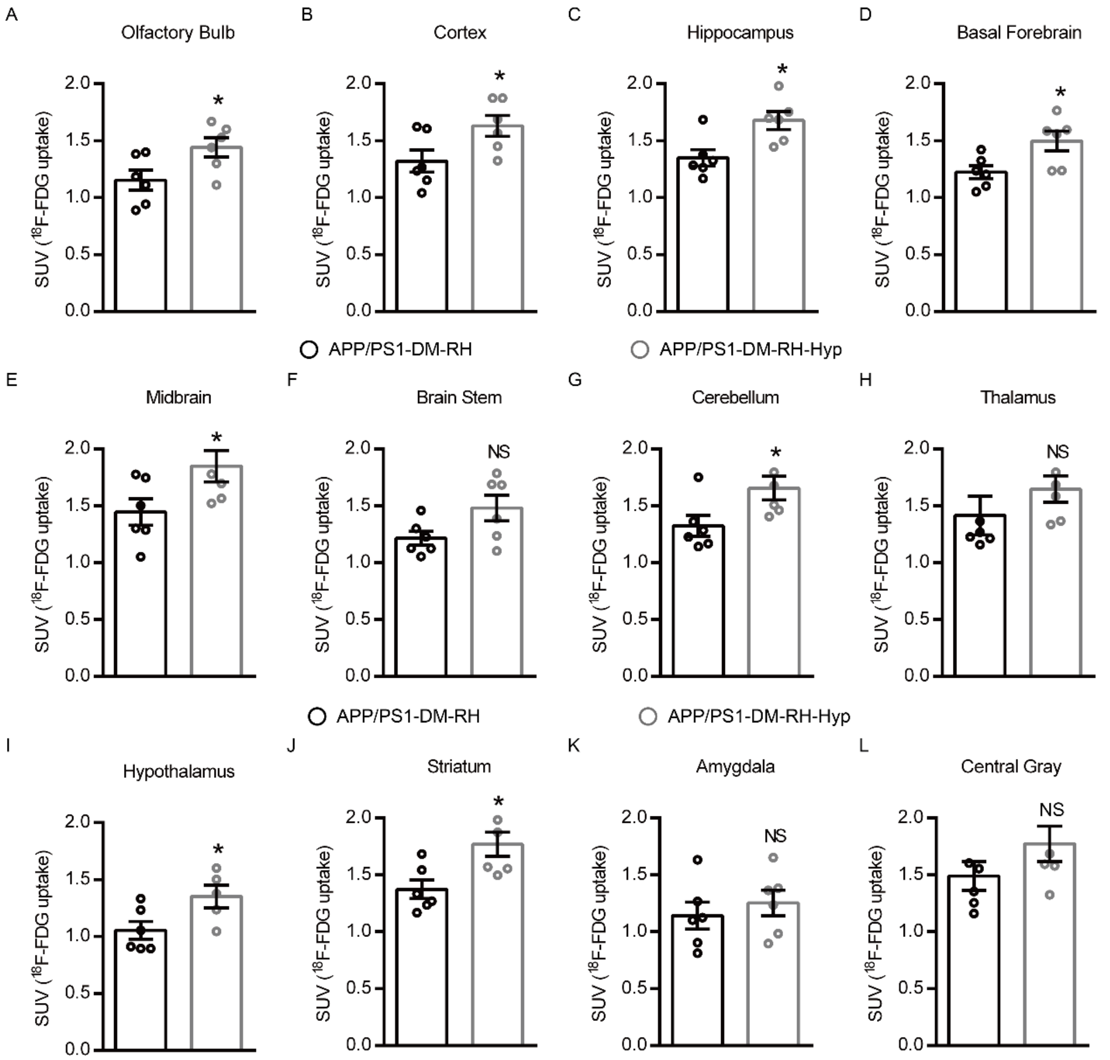

Figure S10, Related to Figure 7. Whole brain ^{18}F-FDG uptake. (A-L) The standard uptake value (SUV) of ^{18}F-FDG in different brain regions measured by ^{18}F-FDG PET/CT scanning (n=6 mice for each group). The data are expressed as the mean ± SEM. Statistical significance was assessed using unpaired student's t test. $^{*}P<0.05$ and $^{**}P<0.01$; NS, no significant difference.

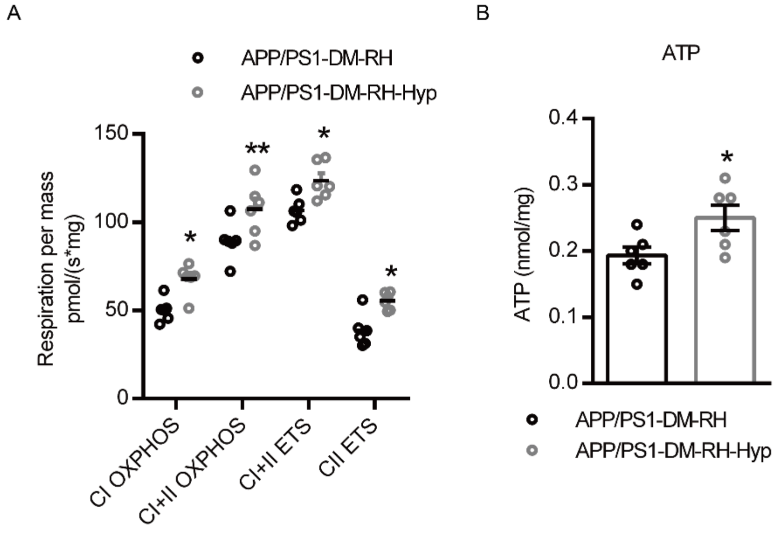

Figure S11, Related to Figure 7. Hyperforin treatment improves mitochondrial function. (A) High-resolution respirometry measured the oxygen consumption capacity of hippocampal mitochondria (n=6 for each group). **(B)** ATP content in hippocampus (n=6 for each group). The data are expressed as the mean ± SEM. Statistical significance was assessed using unpaired student's t test (panel B) or two-way ANOVA followed by Sidak's multiple comparisons test (panel A). **P*＜0.05, ***P*＜0.01.

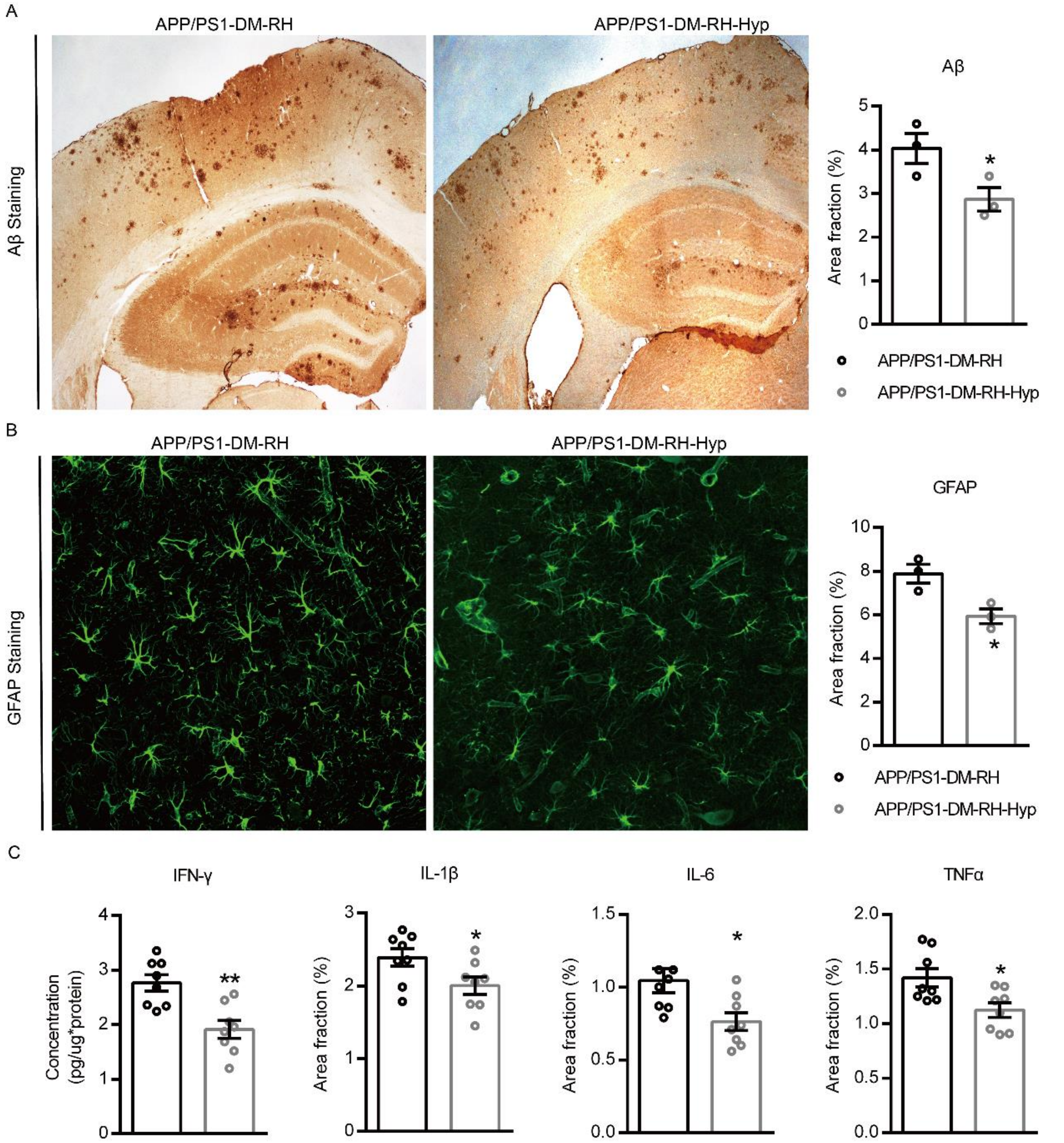

Figure S12, Related to Figure 7. Hyperforin treatment improves AD-type pathologies. (A) Representative images of Aβ immunohistochemistry staining (n=3 mice for each group). Hyp, hyperforin. **(B)** Representative images of GFAP immunofluorescent staining (n=3 mice for each group). **(C)** Quantification of IFN-γ、IL-1β、IL-6 and TNFα by ELISA in hippocampal homogenates (n=8 mice for each group). The data are expressed as the mean ± SEM. Statistical significance was assessed using unpaired student's t test. *$P < 0.05$ and **$P < 0.01$; NS, no significant difference.

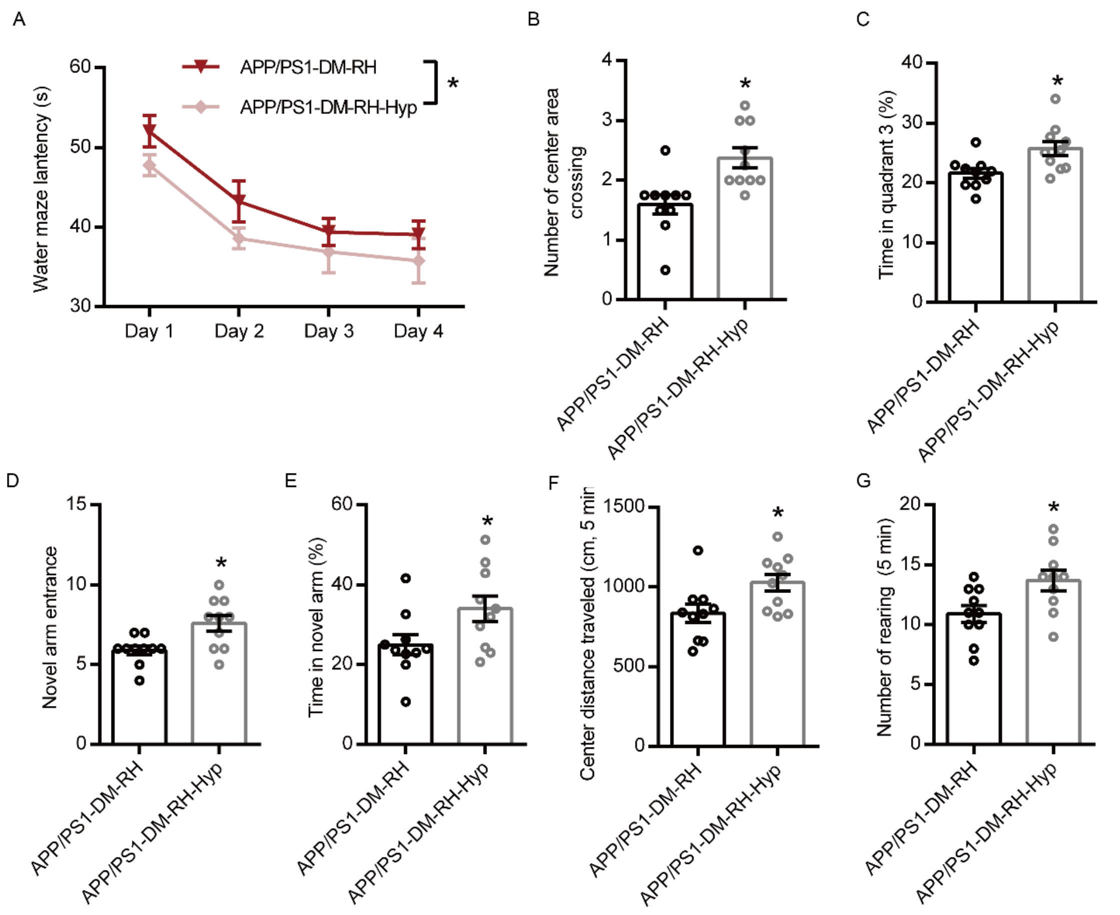

Figure S13, Related to Figure 7. The performances of mice treated with hyperforin in behavioral tests. (A-C) Escape latency during platform trials (A), number of center area crossing (B) and time spend in quadrant 3 o (Q3, C) in probe test. **(D and E)** Novel arm entrance (D) and time spend in the novel arm (E) in Y-maze test. **(F-G)** Distance traveled in center region (F) and number of rearing (G). Hyp, hyperforin, a TRPC6 agonist. n=10 mice for each group. The data are expressed as the mean ± SEM. Statistical significances were assessed using two-way ANOVA followed (panel A) or unpaired student's t test (panel B-G). $^{*}P<0.05$.

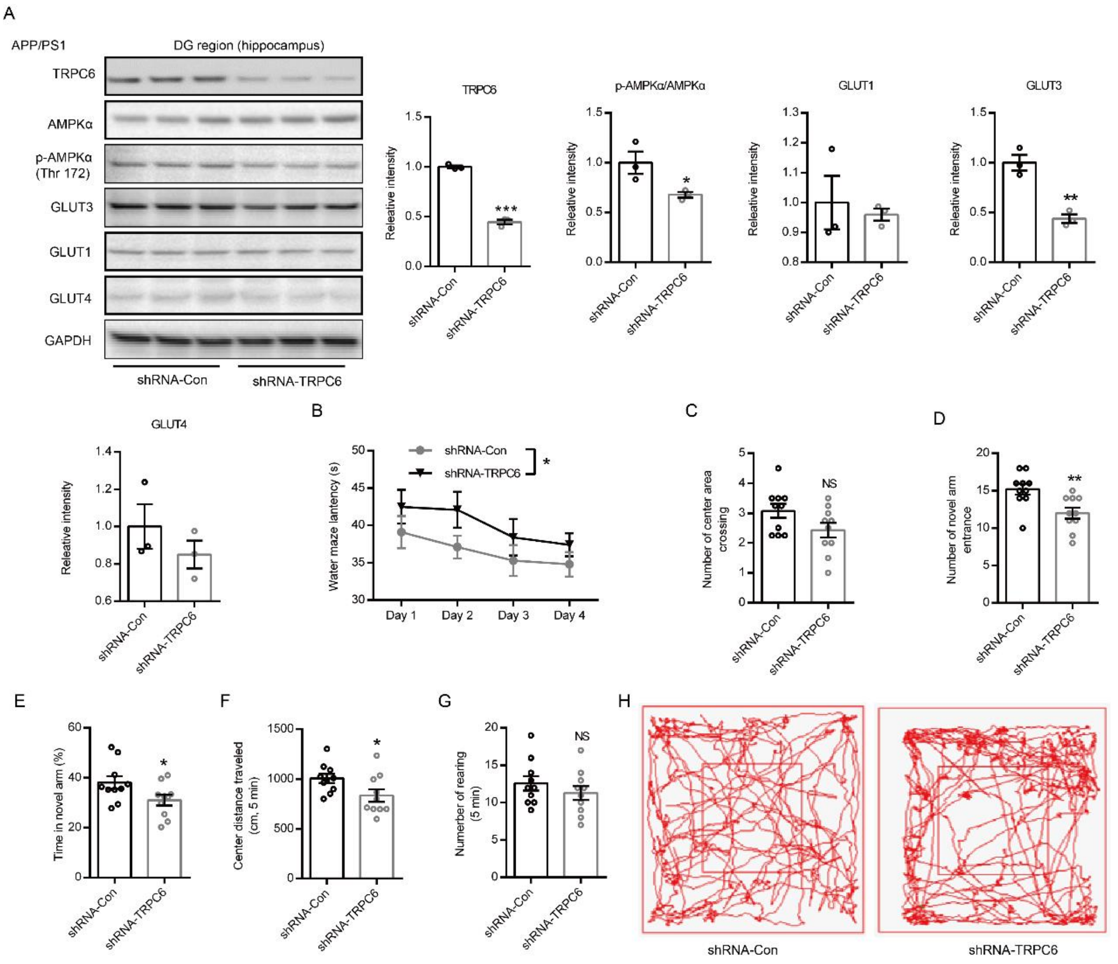

Figure S14, Related to Figure 7. TRPC6 down-regulation with shRNA. (A) Western-blot show the expression of TRPC6, AMPKα，p-AMPKα (Thr172)，GLUT3，GLUT1 and GLUT4 in DG hippocampal region. Quantitative data are shown on the right (n=3). shRNA-Con, APP/PS1 mice were bilaterally injected with empty AAV2/9 virus into DG area of hippocampus; shRNA-TRPC6, APP/PS1 mice were bilaterally injected with AAV2/9 virus expressing mouse shRNA (TRPC6) into DG area of hippocampus. **(B and C)** Escape latency during platform trials (B), number of center area crossing (C) in Morris water-maze test. **(D and E)** Number of novel arm entrance (D) and time spend in the novel arm (E) in Y-maze test. **(F-H)** Distance traveled in center region (F), number of rearing (G) and representative tracing graphs in open field test. n=10 mice for each group. The data are expressed as the mean ± SEM. Statistical significances were assessed using two-way ANOVA followed (panel B) or unpaired student's t test (panel A, C-G). $^{*}P<0.05$, $^{**}P<0.01$.

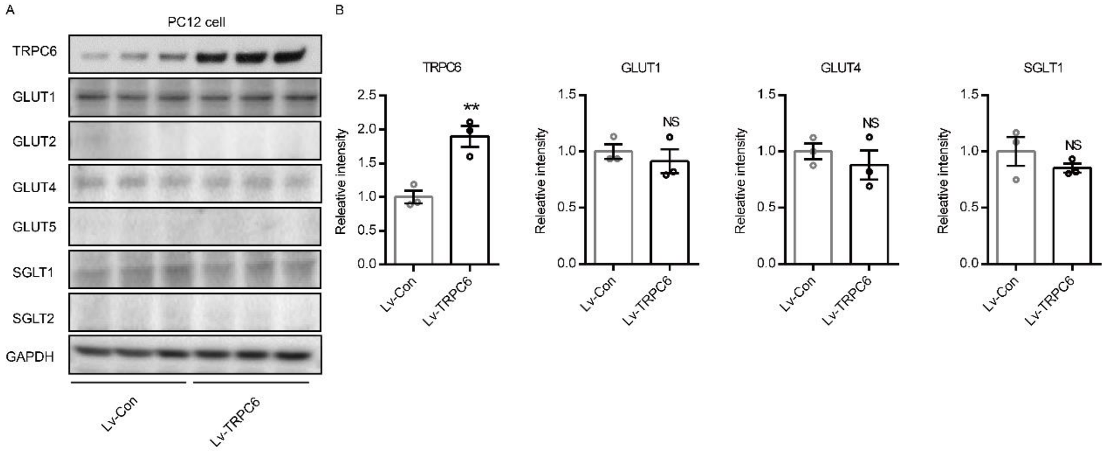

Figure S15, Related to Figure 7. GLUTs and SGLT1/2 expression. (A and B) Western blot and quantitation for TRPC6, GLUT1, GLUT2, GLUT4, GLUT5, SGLT1 and SGLT2 expression in PC12 cells. Lv-Con, the recombinant lentiviral vector; Lv-TRPC6, the recombinant lentiviral vector with TRPC6 over-expression. The data are expressed as the mean ± SEM. Statistical significance was assessed using unpaired student's t test. *$P < 0.05$; NS, no significant difference.

Full unedited gel for Figure 2B

Figure 2B

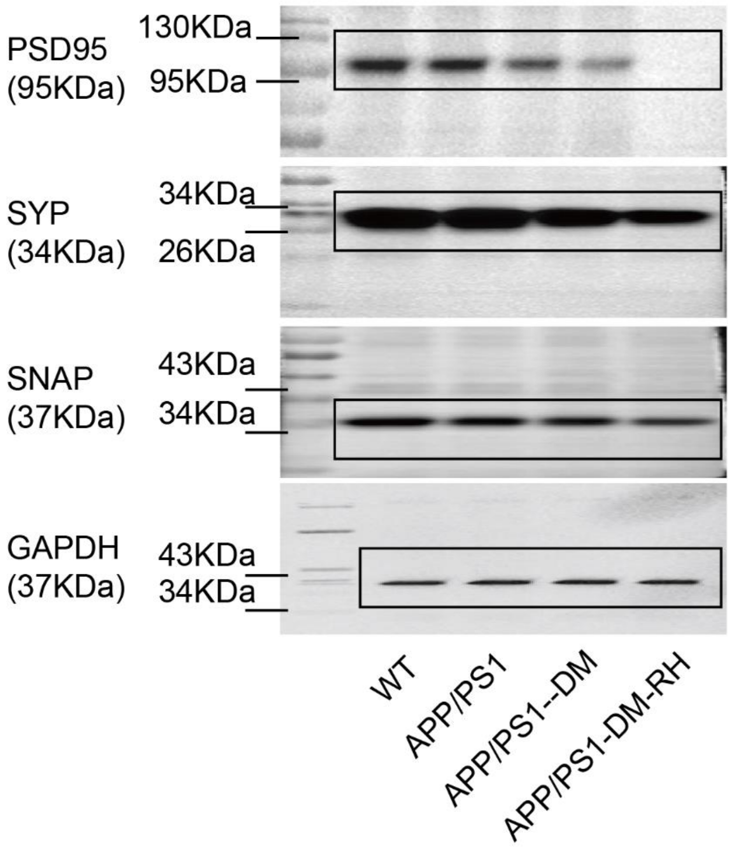

Full unedited gel for Figure 4C

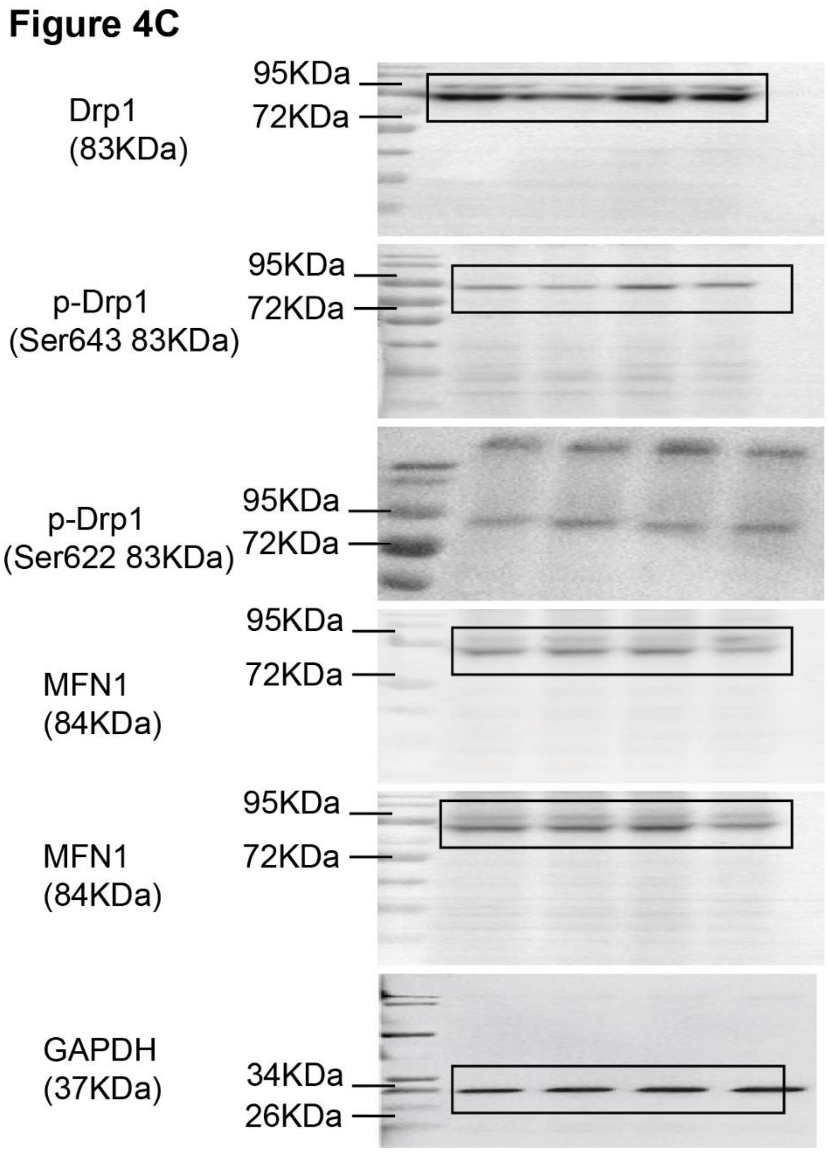

Full unedited gel for Figure 5C

Figure 5C

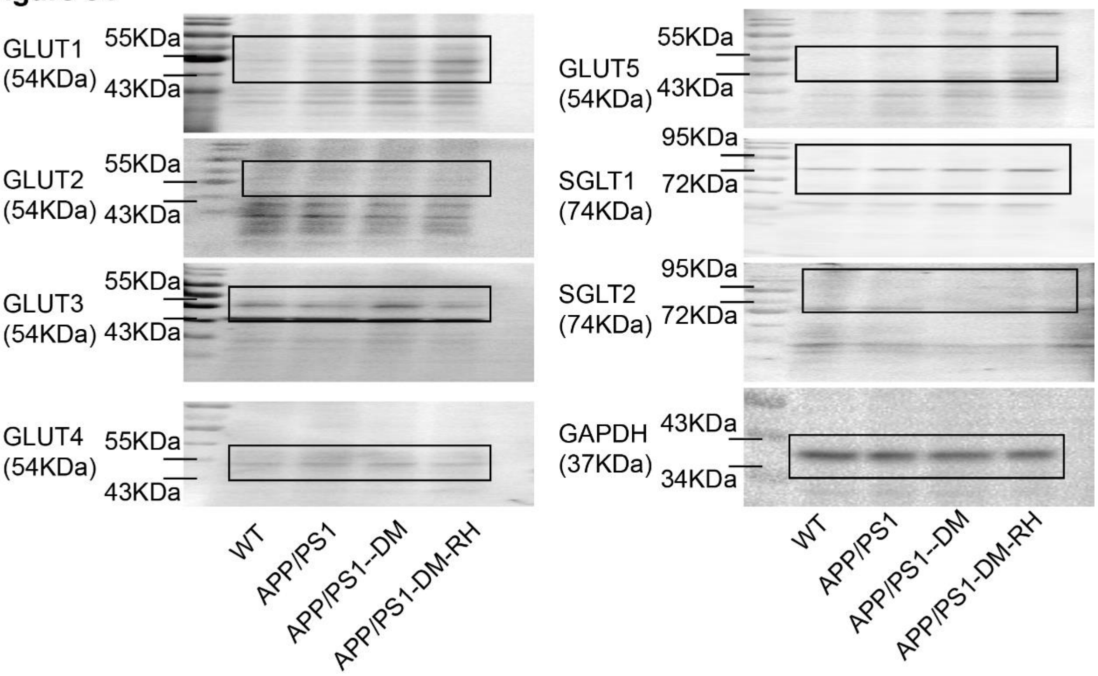

Full unedited gel for Figure 7

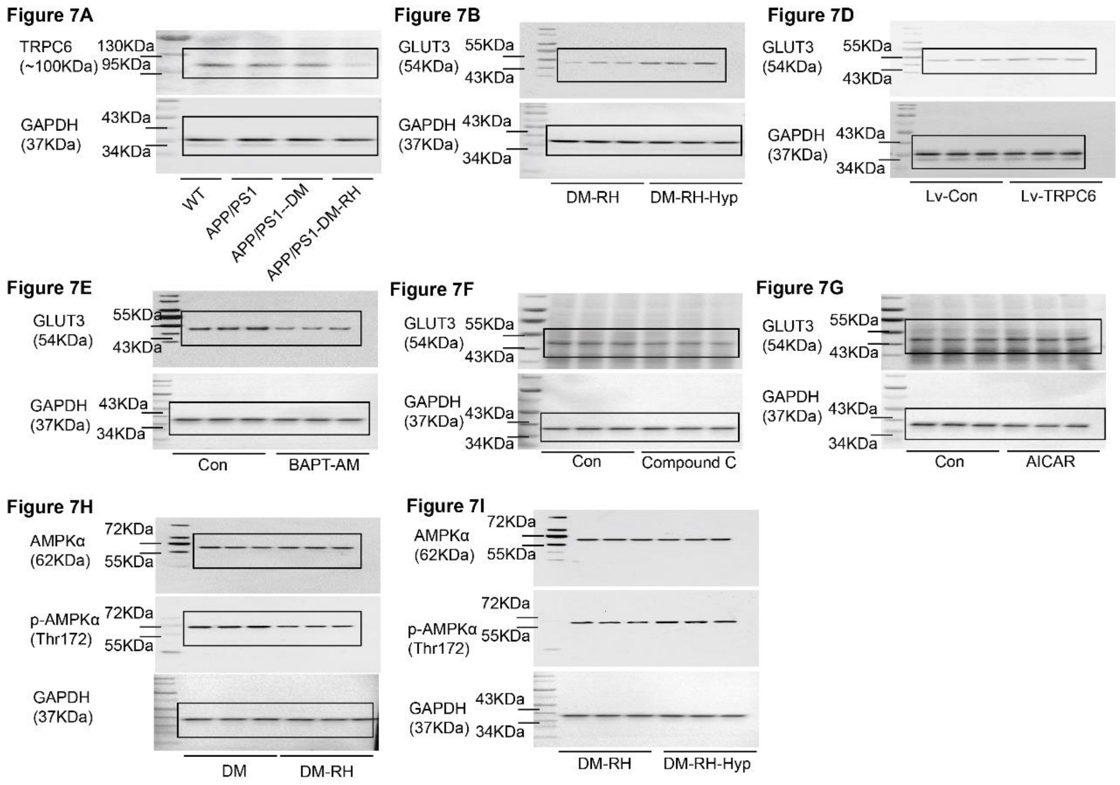

Full unedited gel for Figure S5A

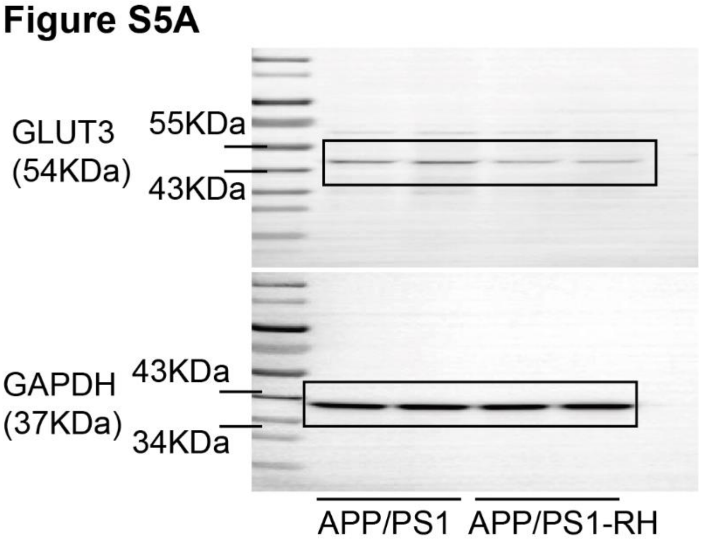

Full unedited gel for Figure S7A

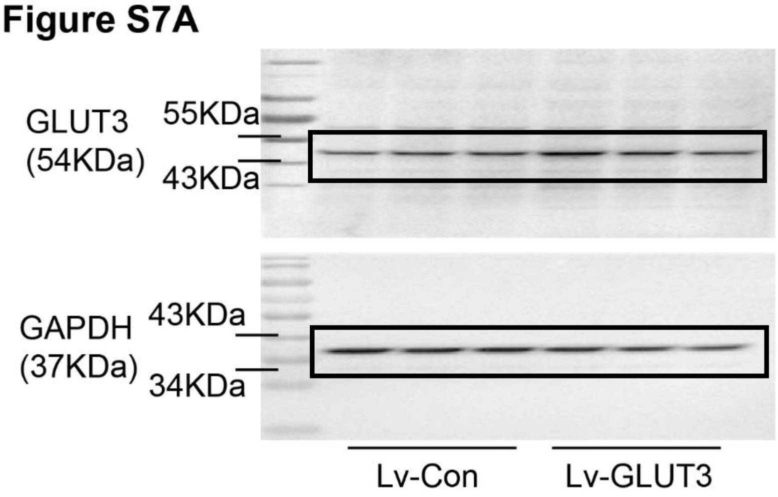

Full unedited gel for Figure S8

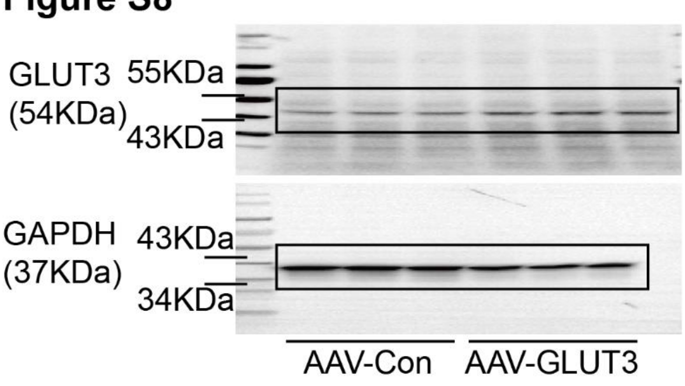

Full unedited gel for Figure S14

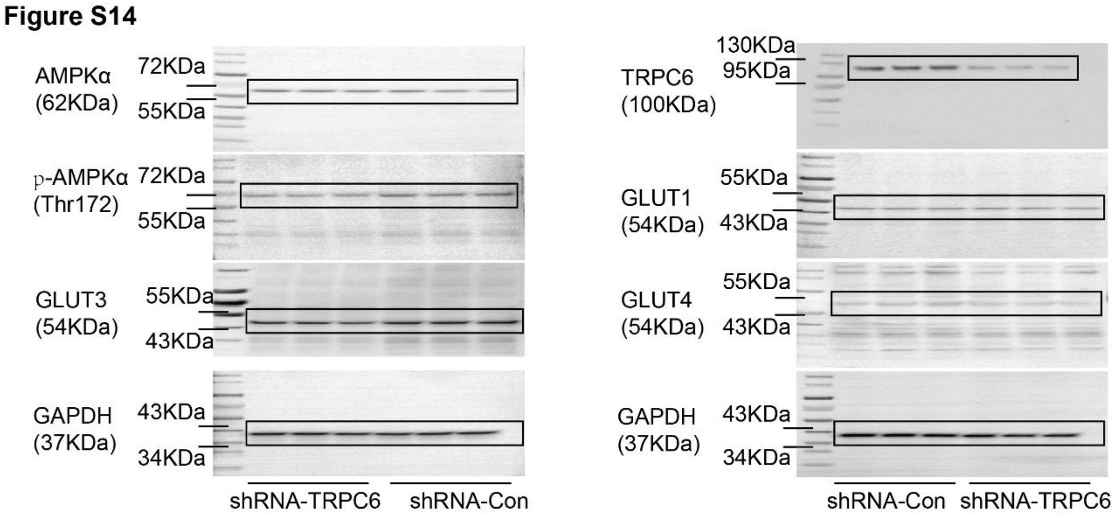

Full unedited gel for Figure S15

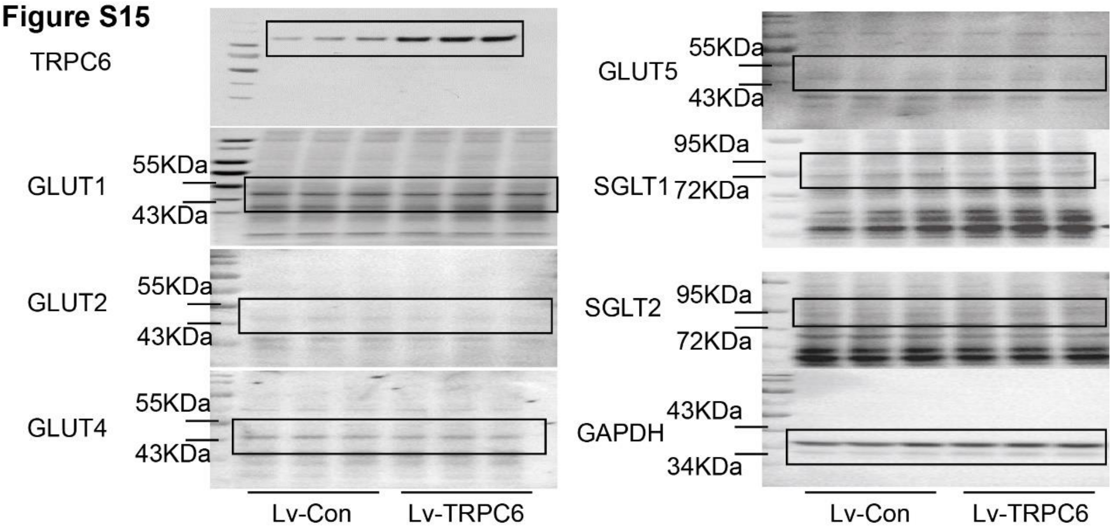

Supplement: Supplemental data [file jciinsight-7-154595-s159.pdf]
